# TLR8 agonist selgantolimod regulates Kupffer cell differentiation status and impairs HBV entry into hepatocytes via an IL-6-dependent mechanism

**DOI:** 10.1136/gutjnl-2023-331396

**Published:** 2024-05-02

**Authors:** Armando Andres Roca Suarez, Marie-Laure Plissonnier, Xavier Grand, Maud Michelet, Guillaume Giraud, Maria Saez-Palma, Anaëlle Dubois, Sarah Heintz, Audrey Diederichs, Nicolaas Van Renne, Thomas Vanwolleghem, Stephane Daffis, Li Li, Nikita Kolhatkar, Yao-Chun Hsu, Jeffrey J Wallin, Audrey H Lau, Simon P Fletcher, Michel Rivoire, Massimo Levrero, Barbara Testoni, Fabien Zoulim

**Affiliations:** 1 INSERM U1052, CNRS UMR-5286, Cancer Research Center of Lyon (CRCL), Lyon, France; 2 University of Lyon, Université Claude-Bernard (UCBL), Lyon, France; 3 The Lyon Hepatology Institute EVEREST, Lyon, France; 4 Viral Hepatitis Research Group, Laboratory of Experimental Medicine and Pediatrics, Antwerp University, Antwerp, Belgium; 5 Department of Gastroenterology and Hepatology, Antwerp University Hospital, Antwerp, Belgium; 6 Gilead Sciences Inc, 324 Lakeside Dr, Foster City, CA, USA; 7 Center for Liver Diseases, E-Da Hospital/I-Shou University, Kaohsiung, Taiwan; 8 INSERM U1032, Centre Léon Bérard (CLB), Lyon, France; 9 Department of Hepatology, Croix Rousse hospital, Hospices Civils de Lyon, Lyon, France; 10 Department of Internal Medicine - DMISM and the IIT Center for Life Nanoscience (CLNS), Sapienza University, Rome, Italy

**Keywords:** KUPFFER CELL, HEPATITIS B, IMMUNOTHERAPY

## Abstract

**ABSTRACT:**

**Objective:**

Achieving HBV cure will require novel combination therapies of direct-acting antivirals and immunomodulatory agents. In this context, the toll-like receptor 8 (TLR8) agonist selgantolimod (SLGN) has been investigated in preclinical models and clinical trials for chronic hepatitis B (CHB). However, little is known regarding its action on immune effectors within the liver. Our aim was to characterise the transcriptomic changes and intercellular communication events induced by SLGN in the hepatic microenvironment.

**Design:**

We identified *TLR8*-expressing cell types in the human liver using publicly available single-cell RNA-seq data and established a method to isolate Kupffer cells (KCs). We characterised transcriptomic and cytokine KC profiles in response to SLGN. SLGN’s indirect effect was evaluated by RNA-seq in hepatocytes treated with SLGN-conditioned media (CM) and quantification of HBV parameters following infection. Pathways mediating SLGN’s effect were validated using transcriptomic data from HBV-infected patients.

**Results:**

Hepatic *TLR8* expression takes place in the myeloid compartment. SLGN treatment of KCs upregulated monocyte markers (eg, *S100A12*) and downregulated genes associated with the KC identity (eg, *SPIC*). Treatment of hepatocytes with SLGN-CM downregulated *NTCP* and impaired HBV entry. Cotreatment with an interleukin 6-neutralising antibody reverted the HBV entry inhibition.

**Conclusion:**

Our transcriptomic characterisation of SLGN sheds light into the programmes regulating KC activation. Furthermore, in addition to its previously described effect on established HBV infection and adaptive immunity, we show that SLGN impairs HBV entry. Altogether, SLGN may contribute through KCs to remodelling the intrahepatic immune microenvironment and may thus represent an important component of future combinations to cure HBV infection.

**WHAT IS ALREADY KNOWN ON THIS TOPIC**

**WHAT THIS STUDY ADDS**

- SLGN triggers Kupffer cell (KC) plasticity to promote the expression of antiviral programmes over those associated with the KC identity.
- SLGN-treated KCs communicate with hepatocytes via interleukin 6, instructing them to reduce NTCP levels, thereby rendering them less susceptible to HBV infection.

**HOW THIS STUDY MIGHT AFFECT RESEARCH, PRACTICE OR POLICY**

- By showing an additional mode of action of SLGN on viral entry and KC remodelling, this study has relevant implications for the clinical development of novel combination therapies for CHB and the identification of intrahepatic biomarkers associated with innate immune activation.
- Our work underscores the importance of considering potential cell states when annotating hepatic populations based on single-cell transcriptomic data.

## Introduction

 Chronic hepatitis B (CHB) affects close to 300 million people worldwide, and is one of the major causes for the development of cirrhosis and hepatocellular carcinoma.^1^ The current treatment regimens based on nucleos(t)ide analogues require indefinite treatment to maintain viral suppression and prevent the virological relapse that usually occurs after discontinuation.^2^ Moreover, current therapeutic options rarely achieve eradication of the virus and early data suggest that new direct-acting antivirals alone are insufficient to restore effective immune control of the infection.^3^ This has led to a renewed interest in the development of novel host-targeting agents aimed to stimulate and reinvigorate immune responses against HBV.^4^

A therapeutic approach currently in clinical development for CHB is to induce activation of innate immune components with toll‐like receptor (TLR) agonists, which may result in both direct and indirect antiviral effects. In this context, the TLR7 agonist vesatolimod was previously developed as a potential immunomodulatory strategy.^5^ Despite promising results in animal models of HBV infection, vesatolimod failed to show therapeutic efficacy in patients with CHB.^6 7^ More recently, the selective toll-like receptor 8 (TLR8) agonist selgantolimod (SLGN) was shown to be safe and well-tolerated, inducing reductions in hepatitis B surface antigen (HBsAg) and hepatitis B e antigen (HBeAg) levels in early phase clinical trials that support its development as a component of future HBV cure regimens.^8 9^ Its mode of action is supposed to be based on the stimulation of multiple pathways and cytokines. These cytokines produced by human peripheral blood mononuclear cells in response to SLGN were shown to reduce viral parameters in HBV‐infected primary human hepatocytes (PHH).^10^ Moreover, SLGN is able to indirectly modulate the action of multiple immune effectors via cytokines produced in *TLR8*-expressing cells. Some of these include an improved cytolytic and non-cytolytic function of natural killer (NK) cells, reduced frequency of regulatory T cells and increased frequency of follicular helper CD4^+^ T cells.^11^

Although these studies provide important insights into the immunomodulatory effect of SLGN in the peripheral compartment, little is known regarding its effect on the immune responses in the liver. With the aim of reshaping the liver immune microenvironment to induce a functional cure of HBV infection, there are several concepts to consider. First, the liver harbours specialised cell populations, such as the transcriptionally distant liver‐resident NK cells, memory CD8^+^ T cells and Kupffer cells (KCs),^12^,^15^ which may play important roles in the immune control of infection. Second, the hepatic microenvironment presents many mechanisms aimed to ensure the suppression of immune responses, often resulting in tolerance.^16^ Third, understanding the intrahepatic pathways associated with effective antiviral immune responses could help identifying biomarkers for patient stratification and/or treatment monitoring during the clinical development of immune interventions.^17^

Accordingly, in this study, we have evaluated the immunomodulatory effects of SLGN on human KCs and their subsequent antiviral effect. We also provide insight on its underlying mechanism of action through intercellular communication events between KCs and HBV-infected hepatocytes.

## Methods

### Primary cell isolation and culture

After a two-step collagenase perfusion, the liver extract was filtered and centrifuged, as previously described.^18^ PHH were cultured on a collagen layer and maintained in Williams E medium (Gibco, Billings, MT, USA) supplemented with 5% FCII serum (Cytiva, Marlborough, MA, USA), 50 U/mL of penicillin/streptomycin, 5 µg/mL of bovine insulin, 2% DMSO (Sigma-Aldrich, St. Louis, MO, USA), 1x Glutamax (Gibco) and 5×10^−5^ M of hydrocortisone (SERB, Brussels, BE). KCs were purified from the non-parenchymal cell mixture by a two-phase iodixanol gradient (Optiprep, BioVision, Waltham, MA, USA), followed by positive selection with the CD163 MicroBead kit (Miltenyi Biotec, Bergisch Gladbach, DE). KCs were seeded at 3×10^5^ cells/well into 24-well plates and cultured in RPMI-1640 medium (Thermo Fisher Scientific, Waltham, MA, USA) supplemented with 10% FCII serum and 50 U/mL of penicillin/streptomycin. HepaRG cells were cultured and DMSO-differentiated as previously described.^19^ All cells were cultured at 37°C in a humidified 5% CO_2_ incubator.

### KC treatment and characterisation of conditioned media

KCs or monocyte-derived macrophages (MDMs) were stimulated with 150 nM SLGN (Gilead Sciences, Foster City, CA, USA) or DMSO for 24 hours to produce SLGN-conditioned media (CM) and Mock-CM, respectively. Cell supernatants were collected and stored at −80°C. Cytokine profiles of the CM were characterised by Luminex assay using a custom ProcartaPlex panel (Invitrogen, Waltham, MA, USA) and a MAGPIX instrument, according to the manufacturer’s instructions. Interleukin (IL)-24 and oncostatin M (OSM) levels were quantified in the CM by ELISA (R&D Systems, Minneapolis, MN, USA). Multiple batches of CM were used, all of which had comparable cytokine profiles. Additionally, KCs were treated with 250 ng/mL of lipopolysaccharides (LPS, Sigma-Aldrich) for 24 hours in order to perform gene expression analyses.

### HBV infection and cytokine neutralisation assay

PHH or HepaRG cells were treated with Mock-CM or SLGN-CM (1/50) for 72 hours, with media been changed daily. Cells were subsequently infected with HBV (PEG-precipitated, 500 viral genome equivalents/cell) in the presence of CM, as previously described.^20^ Media was replaced every 2 days (no CM) until the end of the infection period (6 days). Bulevirtide (100 nM, 24 hours) was used as HBV entry control (n=5). Cell viability was determined using the CellTiter-Glo 2.0 assay (Promega, Madison, WI, USA). For the cytokine neutralisation assay, PHH were treated with neutralising antibodies against IL-6, OSM, IL-24 and IL-1α or isotype controls (1 µg/mL) in the presence of CM before HBV infection (online supplemental table 1). Treatment with recombinant IL-6 (5 ng/mL), OSM, IL-24 and IL-1α (5 pg/mL and 5 ng/mL) were used as positive controls (Miltenyi Biotec).

### Bulk RNA-seq

KCs treated with SLGN (150 nM, 24 hours, n=3) or DMSO, and PHH treated with SLGN-CM or Mock-CM (1/50, 72 hours, n=3) were lysed with TRI Reagent, followed by RNA extraction using the Direct-zol RNA Miniprep kit (Zymo Research, Irvine, CA, USA), according to the manufacturer’s instructions. Libraries were prepared using the TruSeq Stranded mRNA Sample Preparation kit (Illumina, San Diego, CA, USA) and sequenced on a NovaSeq 6000 as 42-nt paired-end reads (Active Motif, Carlsbad, CA, USA).

### Bioinformatics analyses

For the analysis of bulk RNA-seq data, raw reads were preprocessed using fastp (V.0.23.2) and then mapped using hisat2 (V.2.2.1) to the human reference genome (GRCh38.99).^21 22^ Mapped reads were filtered using samtools (V.1.11) and the number of reads per gene was counted using HTSeq (V.0.13.5).^23 24^ Differential expression analysis was carried out with the DESeq2 package (V.1.1.0).^25^ Quality check of raw and preprocessed reads was performed using fastqc (V.0.11.5) and compiled using multiqc (V.1.11).^26 27^ Volcano plots were generated using the EnhancedVolcano R package (V.1.14). Signalling pathway activities were estimated by gene set enrichment analysis (GSEA, V.20.4) using all gene sets from the Molecular Signatures Database (V.2023).^28 29^ NicheNet ligand activity prediction (V.2) was performed using all expressed KC and hepatocyte genes (mean log_2_ value >2) and the differentially expressed genes in response to SLGN-CM (FDR<0.05) as gene set of interest.^30^

Single-cell liver transcriptomic data and code for the analyses were obtained from the GEO accession GSE192742 and www.livercellatlas.org.^14^ Expression levels of the SLGN-DOWN KC signature were estimated using gene set variation analysis (GSVA, V.1.44.5).^31^ Graphs were generated using the *VlnPlot* and *DotPlot* functions of Seurat (V.4.9).^32^

### Statistical analyses

All statistical analyses were performed in Prism (V.10, Dotmatics, Boston, MA, USA). Tests used are indicated in the figure legends (Mann-Whitney test, Kruskal‐Wallis with Dunn’s multiple comparison test, analysis of variance with Dunnett’s multiple comparison test, Spearman correlation, Wald test).

### Additional materials and methods

Methods and reagents for immunofluorescence studies, RT-qPCR, chromatin immunoprecipitation (ChIP)-qPCR, generation of MDMs, western blot, detection of HBV antigens, cynomolgus macaque experiments, human liver biopsy sample processing and additional bioinformatics analyses are provided in online supplemental material.

## Results

### Hepatic *TLR8* expression takes place primarily in the myeloid compartment

**Figure 1:**
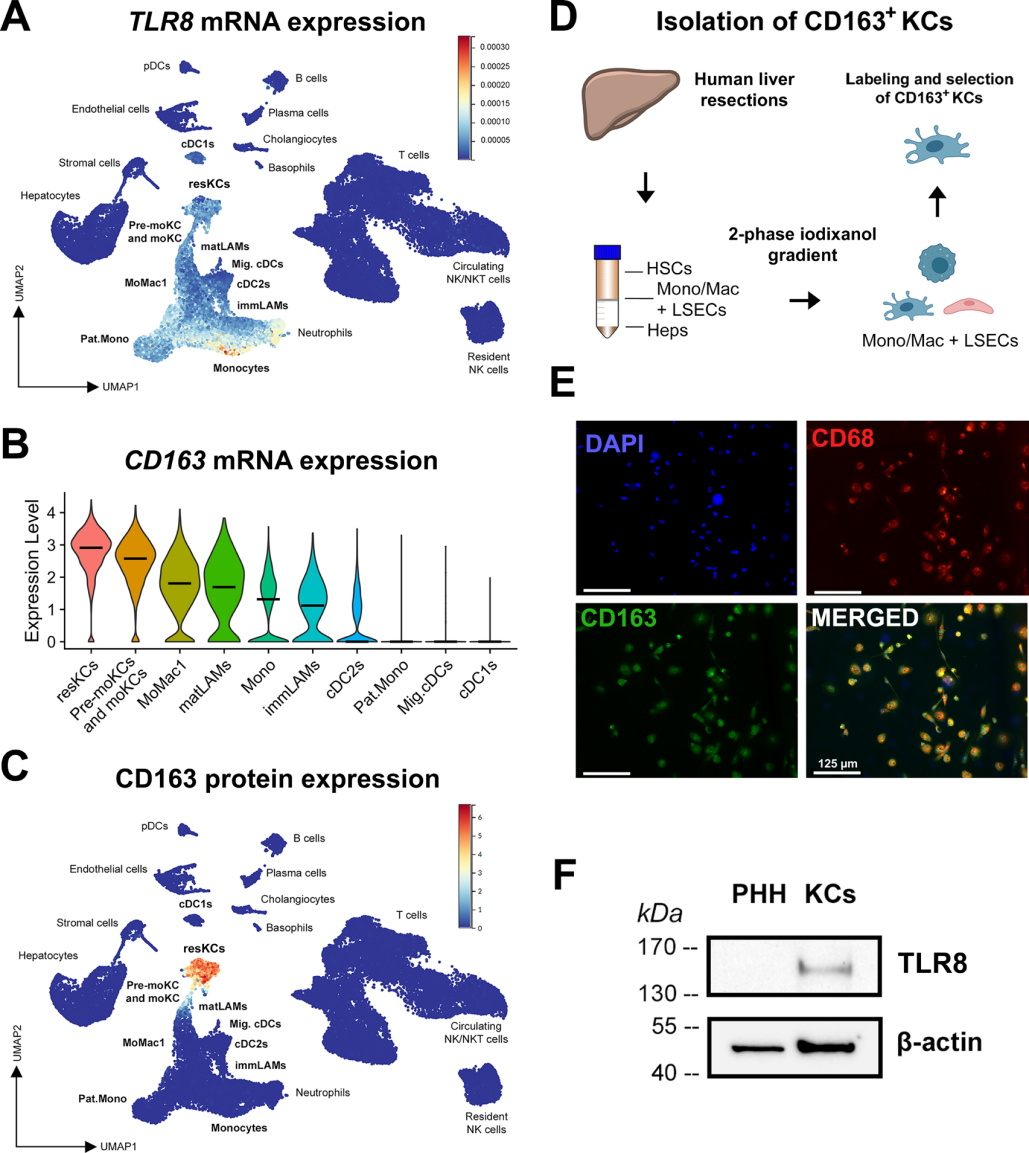
Hepatic *TLR8* expression takes place primarily in the myeloid compartment. (**A**) Expression of *TLR8* mRNA in each cell population of the human liver microenvironment (GSE192742). (**B**) Expression of *CD163* mRNA in the myeloid compartment. Violin plots represent mean expression values. (**C**) Expression of CD163 protein in each cell population of the human liver microenvironment. (**D**) KC isolation method based on a two-phase iodixanol gradient and CD163-positive selection. (**E**) Immunofluorescence image of KCs isolated with the protocol described in (**D**), showing positive staining for nuclear DNA (DAPI, blue), CD68 (red) and CD163 (green). (**F**) Western blot showing expression of TLR8 protein in human KCs as compared with PHH from the same donor. cDCs, classical dendritic cells; HSCs, hepatic stellate cells; KCs, Kupffer cells; Mig.cDCs, migratory cDCs; moKCs, monocyte-derived KCs; NK cells, natural killer cells; LAMs, lipid-associated macrophages; LSECs, liver sinusoidal endothelial cells; Pat.Mono, patrolling monocytes; pDCs, plasmacytoid dendritic cells; PHH, primary human hepatocytes; TLR8, toll-like receptor 8.

To characterise the intrahepatic response to SLGN, we first identified cell populations that express *TLR8* in the human liver microenvironment and are therefore, susceptible to the direct action of this agonist. The analysis of publicly available scRNA-seq data obtained from healthy human donors,^14^ showed that hepatic *TLR8* expression takes place mainly in the myeloid compartment (figure 1A). Considering that the liver harbours the most abundant pool of macrophages in the human body and that these cells represent an essential constituent of the mononuclear phagocytic system,^33^ we focused on the characterisation of liver-resident macrophages or KCs. We also took into account how CD163 has been described as a reliable marker for the identification of KCs (figure 1B,C).^14^ Thus, we optimised a protocol based on a two-phase iodixanol gradient and positive selection of CD163-expressing cells from human liver resections (figure 1D). Our results show that this method allows to obtain a homogeneous population of CD163^+^/CD68^+^ human KCs (figure 1E, online supplemental figure 1), which expressed TLR8 and were subsequently used to explore the action of SLGN (figure 1F).

### SLGN treatment leads to morphological and transcriptomic changes associated with KC differentiation status

**Figure 2:**
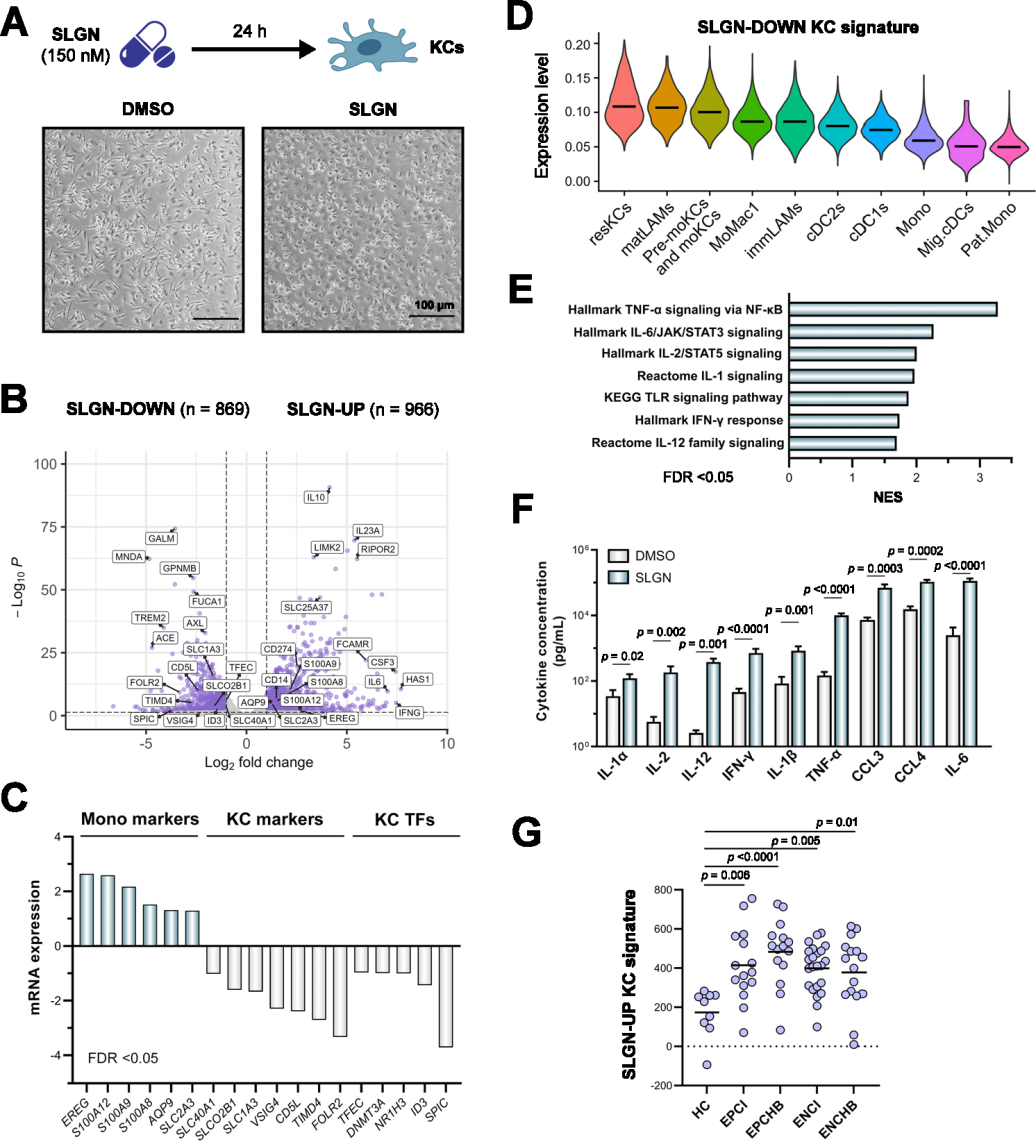
SLGN treatment leads to morphological and transcriptomic changes associated with KC differentiation status. (**A**) Microscopic image of human KCs treated with SLGN (150 nM) or DMSO for 24 hours. (**B**) Volcano plot depicting significantly downregulated (SLGN-DOWN, n=869) and upregulated (SLGN-UP, n=966) KC genes in response to SLGN (150 nM) for 24 hours (n=3, FDR<0.05, log_2_FC>1). (**C**) SLGN treatment of KCs (**B**) induces the upregulation of monocyte markers and the downregulation of KC genes and TFs. (**D**) Expression of the SLGN-DOWN KC signature in each population of the hepatic myeloid compartment (GSE192742). Violin plots represent mean expression values. (**E**) GSEA showing significantly upregulated pathways in KCs treated with SLGN (n=3, FDR<0.05). (**F**) Concentration of cytokines from culture supernatants of KCs stimulated with SLGN (150 nM) or DMSO for 24 hours (n=4, Mann-Whitney test). Bars represent mean±SEM. (**G**) Mean expression levels of the SLGN-UP signature in liver transcriptomic data from HBV-infected patients at different disease stages (Kruskal-Wallis test, GSE230397). AQP9, aquaporin 9; CCL, C-C motif chemokine ligand; CD5L, CD5 molecule like; DNMT3A, DNA methyltransferase 3 alpha; ENCHB, HBeAg-positive chronic hepatitis B; ENCI, HBeAg-negative chronic infection; EPCHB, HBeAg-positive chronic hepatitis B; EPCI, HBeAg-positive chronic infection; EREG, epiregulin; FOLR2, folate receptor beta; GSEA, gene set enrichment analysis; HC, healthy control; ID3, inhibitor of DNA binding 3; IFN, interferon; IL, interleukin; KCs, Kupffer cells; moKCs, monocyte-derived KCs; NES, normalised enrichment score; LAMs, lipid-associated macrophages; S100A, S100 calcium binding protein A; SLC, solute carrier family; SLGN, selgantolimod; SPIC, Spi-C transcription factor; TFs, transcription factors; TFEC, transcription factor EC; TIMD4, T cell immunoglobulin and mucin domain containing 4; TNF-α, tumour necrosis factor alpha; TLR, toll-like receptor; VSIG4, V-set and immunoglobulin domain containing 4.

In order to characterise the direct effect of SLGN in the KC population, we treated these cells with SLGN (150 nM) for 24 hours. In contrast to the control condition, SLGN-treated KCs lost the elongated shape with big processes that characterise their morphology (figure 2A).^34^ In line with this, transcriptomic analysis of SLGN-treated KCs showed a wide variety of gene expression changes (figure 2B), which included the significant (FDR<0.05) upregulation of monocyte markers (eg, *EREG*, *S100A12*), the downregulation of KC markers (eg, *FOLR2*, *TIMD4*) and KC-specific transcription factors (eg, *SPIC*, *ID3*, *NR1H3*) (figure 2C). These were among the genes previously employed to identify the KC population within the myeloid compartment of the human liver (online supplemental figure 2A).^14^ The mapping of SLGN downregulated genes (SLGN-DOWN KC, n=869) in this liver atlas showed that their expression takes place mainly in mature cell types such as KCs and mature lipid-associated macrophages (LAMs), with markedly lower levels in immature populations such as monocytes and immature LAMs (figure 2D). These results were validated in a second scRNA-seq data set obtained from healthy human donors (online supplemental figure S3),^35^ suggesting the ensemble of SLGN-DOWN KC genes to be part of transcriptional programmes progressively acquired as cells mature and adapt to the liver microenvironment.^36^ A similar expression profile was observed in KCs treated with the TLR4 ligand LPS (250 ng/mL, 24 hours). This was also accompanied by an increased expression of programmed cell death-1 ligand 1 (PD-L1, *CD274*) in both SLGN-treated and LPS-treated KCs (online supplemental figure S2B, figure 2B), which is in line with previous observations in SLGN-treated polymorphonuclear myeloid‐derived suppressor cells (PMN‐MDSCs).^11^

In parallel to the changes associated with KC differentiation, SLGN induced activation of a series of pathways related to the inflammatory response, such as nuclear factor kappa B (NF-κB), Janus kinase/signal transducer and activator of transcription (JAK/STAT) and interferon (IFN) (figure 2E), which translated into the production of a wide variety of inflammatory cytokines that included IFN-γ, tumour necrosis factor alpha and IL-6 (figure 2F). Similar cytokine profiles were observed following SLGN treatment of MDMs (online supplemental figure 4). These results are in line with the expression of SLGN upregulated genes (SLGN-UP KC, n=966) in liver transcriptomic data from HBV-infected patients,^37^ showing significant (p<0.0001) higher levels at disease phases characterised by active inflammation, such as HBeAg-positive chronic hepatitis B (figure 2G).

**Figure 3:**
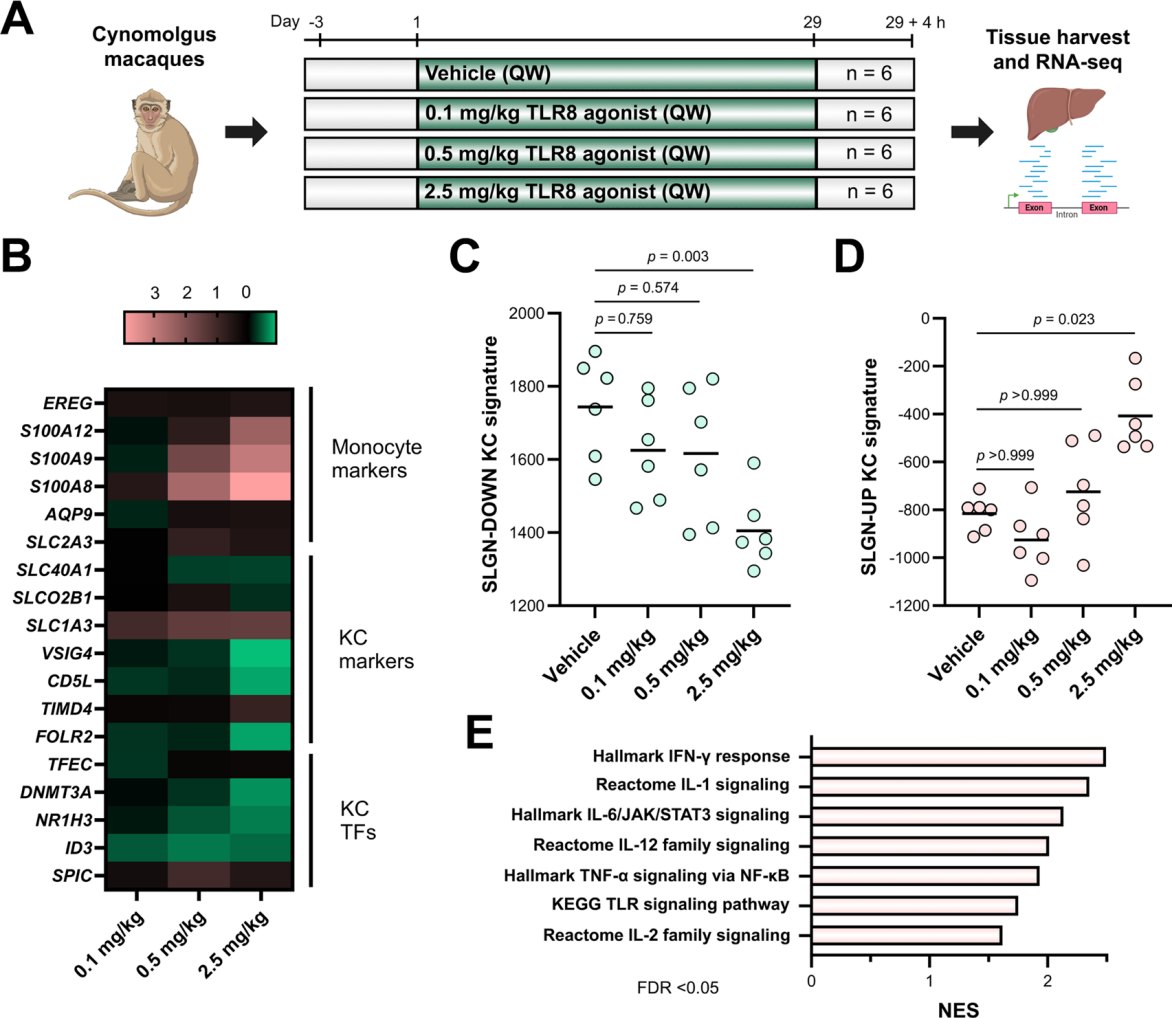
In vivo activation of TLR8 signalling modulates the expression of liver transcriptomic programmes associated with KC differentiation status. (**A**) Cynomolgus macaques (n=6 per group) received weekly doses of TLR8 agonist (0.1, 0.5 and 2.5 mg/kg) during a 4-week period. Liver samples were obtained 4 hours after the last dose in order to perform transcriptomic analyses. (**B**) TLR8 agonist treatment induces the upregulation of monocyte markers and the downregulation of KC genes and TFs in liver tissues from cynomolgus macaques. (**C–D**) Mean expression levels of the (**C**) SLGN-DOWN and (D) SLGN-UP KC signatures in liver tissues of macaques treated with TLR8 agonist (Kruskal-Wallis test). (**E**) GSEA showing significantly upregulated pathways in liver tissues of cynomolgus macaques treated with TLR8 agonist (2.5 mg/kg vs vehicle control, FDR<0.05). AQP9, aquaporin 9; CD5L, CD5 molecule like; DNMT3A, DNA methyltransferase 3 alpha; EREG, epiregulin; FOLR2, folate receptor beta; GSEA, gene set enrichment analysis; ID3, inhibitor of DNA binding 3; KCs, Kupffer cells; NES, normalised enrichment score; S100A, S100 calcium binding protein A; SLC, solute carrier family; SLGN, selgantolimod; SPIC, Spi-C transcription factor; TFs, transcription factors; TFEC, transcription factor EC; TIMD4, T cell immunoglobulin and mucin domain containing 4; TLR8, toll-like receptor 8; VSIG4, V-set and immunoglobulin domain containing 4.

To evaluate the potential modulation of KC differentiation status in vivo, we characterised the effect of TLR8 pathway activation in cynomolgus macaques, a non-human primate in which, similar to humans, the expression of genes belonging to the SLGN-DOWN signature takes place primarily in KCs (online supplemental figure 5). Therefore, animals received weekly doses of TLR8 agonist (0.1, 0.5 and 2.5 mg/kg, n=6 per group) during a 4-week period in order to identify transcriptomic changes taking place within the liver (figure 3A). Our results show that similarly to the profile of SLGN-treated KCs, activation of TLR8 signalling was associated with a dose-dependent downregulation of KC identity genes and the upregulation of monocyte markers (figure 3B). This could also be observed at the gene set level, with a significant decrease of the SLGN-DOWN KC signature (p=0.003), an increase of the SLGN-UP KC signature (p=0.023) and the activation of signalling pathways mediating the inflammatory response (figure 3C–E).

Overall, these results demonstrate that SLGN is able to modulate KC differentiation status in parallel to the induction of an inflammatory response.

### SLGN indirectly downregulates *NTCP* expression and impairs HBV entry into hepatocytes

**Figure 4:**
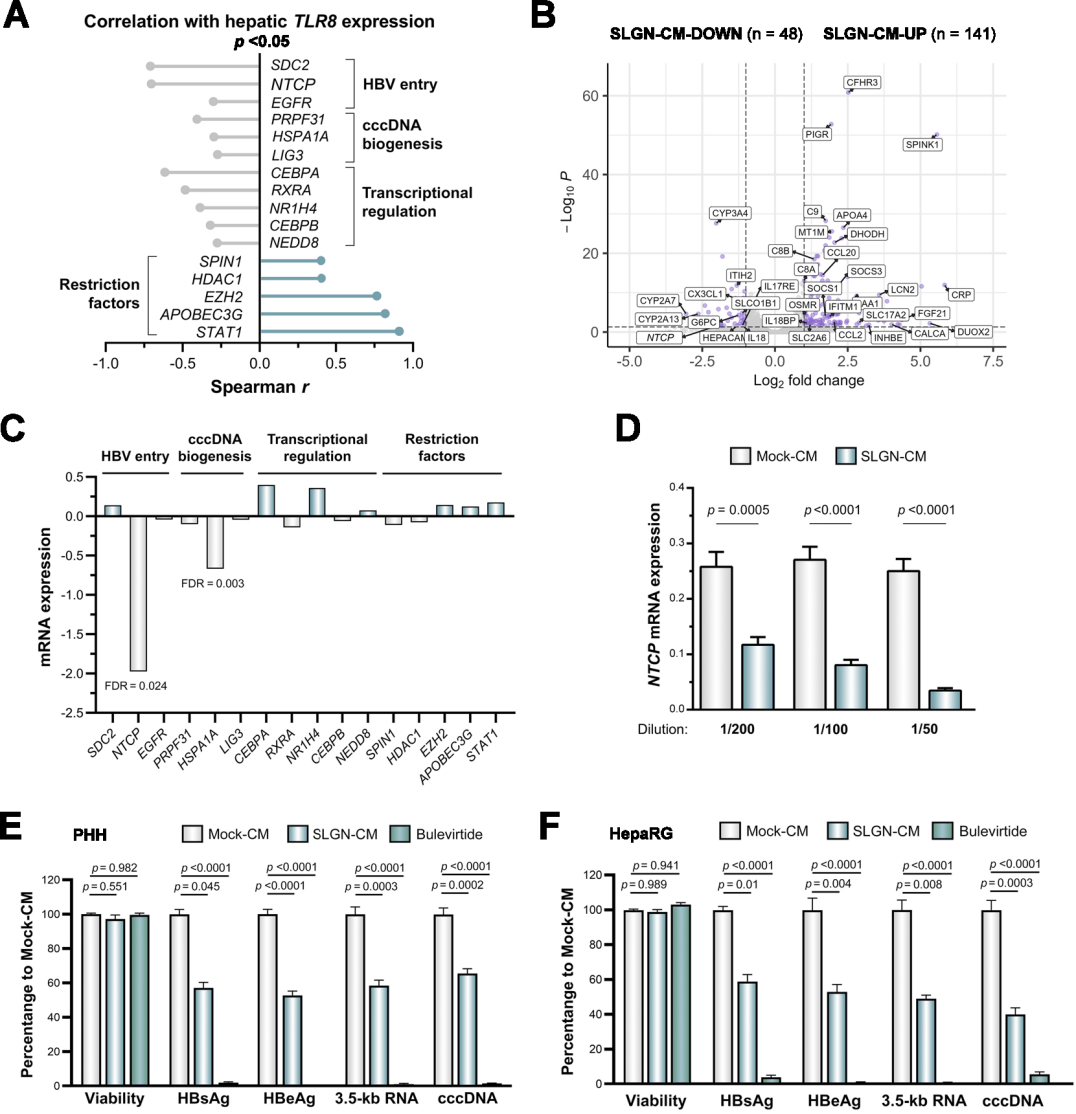
SLGN indirectly downregulates *NTCP* expression and impairs HBV entry into hepatocytes. (**A**) Liver transcriptomic data from HBV-infected patients showing a correlation of *TLR8* expression with genes implicated in the HBV cycle (n=83, Spearman correlation, GSE65359). (**B**) Volcano plot depicting significantly downregulated (SLGN-CM-DOWN, n=48) and upregulated (SLGN-CM-UP, n=141) PHH genes in response to SLGN-CM (n=3, FDR<0.05, FC>1). (**C**) Treatment of PHH with SLGN-CM (1/50, 72 hours) produced in KCs induces the downregulation of *NTCP* and *HSPA1A* (RNA-seq). (**D**) Treatment of PHH with SLGN-CM leads to a dose-dependent downregulation of *NTCP*, as assessed by qPCR (Mann-Whitney test, n=3). Bars represent mean±SEM. (**E–F**) Treatment of PHH (**E**) or differentiated HepaRG cells (**F**) with SLGN-CM (1/50) 72 hours prior HBV infection leads to a significant decrease in HBsAg, HBeAg, 3.5 kb RNA and cccDNA levels at day 6 postinoculation (one-way analysis of variance, n=5). Bulevirtide (100 nM, 24 hours) was used as positive control. Bars represent mean±SEM. APOBEC3G, apolipoprotein B mRNA editing enzyme catalytic subunit 3G; cccDNA, covalently closed circular DNA; CEBPA, CCAAT enhancer binding protein alpha; CM, conditioned media; EGFR, epidermal growth factor receptor; EZH2, enhancer of zeste 2 polycomb repressive complex 2 subunit; HBeAg, hepatitis B e antigen; HBsAg, hepatitis B surface antigen; HDAC1, histone deacetylase 1; HSPA1A, heat shock protein family A (Hsp70) member 1A; NEDD8, NEDD8 ubiquitin-like modifier; NTCP, sodium/taurocholate cotransporting polypeptide; LIG3, DNA ligase 3; PHH, primary human hepatocytes; PRPF31, pre-mRNA processing factor 31; RXRA, retinoid X receptor alpha; SDC2, syndecan 2; SLGN, selgantolimod; SPIN1, spindlin 1; STAT1, signal transducer and activator of transcription 1; TLR8, toll-like receptor 8.

Considering the association of genes belonging to the SLGN-UP KC signature with CHB phases presenting high levels of inflammation, we explored the impact that these KC transcriptional programmes and cytokine profiles induced by SLGN could have on hepatocytes and the HBV cycle. Therefore, we analysed publicly available liver transcriptomic data from HBV-infected patients,^38 39^ by correlating the levels of *TLR8* with those of host factors implicated during HBV infection.^40^ This allowed us to observe a significant (p<0.05) negative correlation between the expression of *TLR8* and genes implicated in HBV entry (ie, *SDC2*, *NTCP* and *EGFR*), cccDNA biogenesis (ie, *PRPF31*, *HSPA1A* and *LIG3*) and HBV transcriptional regulation (ie, *CEBPA*, *RXRA*, *NR1H4*, *CEBPB* and *NEDD8*), while a positive correlation was observed with HBV restriction factors (ie, *SPIN1*, *HDAC1*, *EZH2*, *APOBEC3G* and *STAT1*) (figure 4A, online supplemental figure S6A).

To identify which of these factors are modulated via intercellular communication events between KCs and hepatocytes, we performed a transcriptomic characterisation of PHH treated with SLGN-CM (72 hours, 1/50 dilution) produced in KCs (figure 4B). This showed a significant downregulation of *NTCP* and *HSPA1A* (FDR<0.05) (figure 4C), which was particularly pronounced for *NTCP*, one of the main host factors regulating HBV entry into hepatocytes.^41^ This was further explored in additional PHH donors treated with increasing concentrations of SLGN-CM, showing a dose-dependent downregulation of *NTCP* (figure 4D). The repression of *NTCP* expression most likely occurred at the transcriptional level, as shown by the decrease of phosphorylated polymerase 2 at the *NTCP* promoter in ChIP-qPCR experiments (online supplemental figure 7). Finally, to validate the functional consequence that this SLGN-induced *NTCP* downregulation could have on the HBV cycle, we exposed PHH to SLGN-CM (72 hours) prior HBV infection. This resulted in a significant decrease of viral parameters, comprising HBsAg, HBeAg, 3.5 kb RNA and cccDNA at the end of the 6-day postinoculation observation period (figure 4E). Similar results were obtained in differentiated HepaRG cells (figure 4F). These data suggest that soluble factors produced by KCs in response to SLGN induce a decrease of *NTCP* expression in hepatocytes, which in turn leads to an impaired HBV entry.

### SLGN is able to indirectly activate STAT3 signaling in hepatocytes

**Figure 5:**
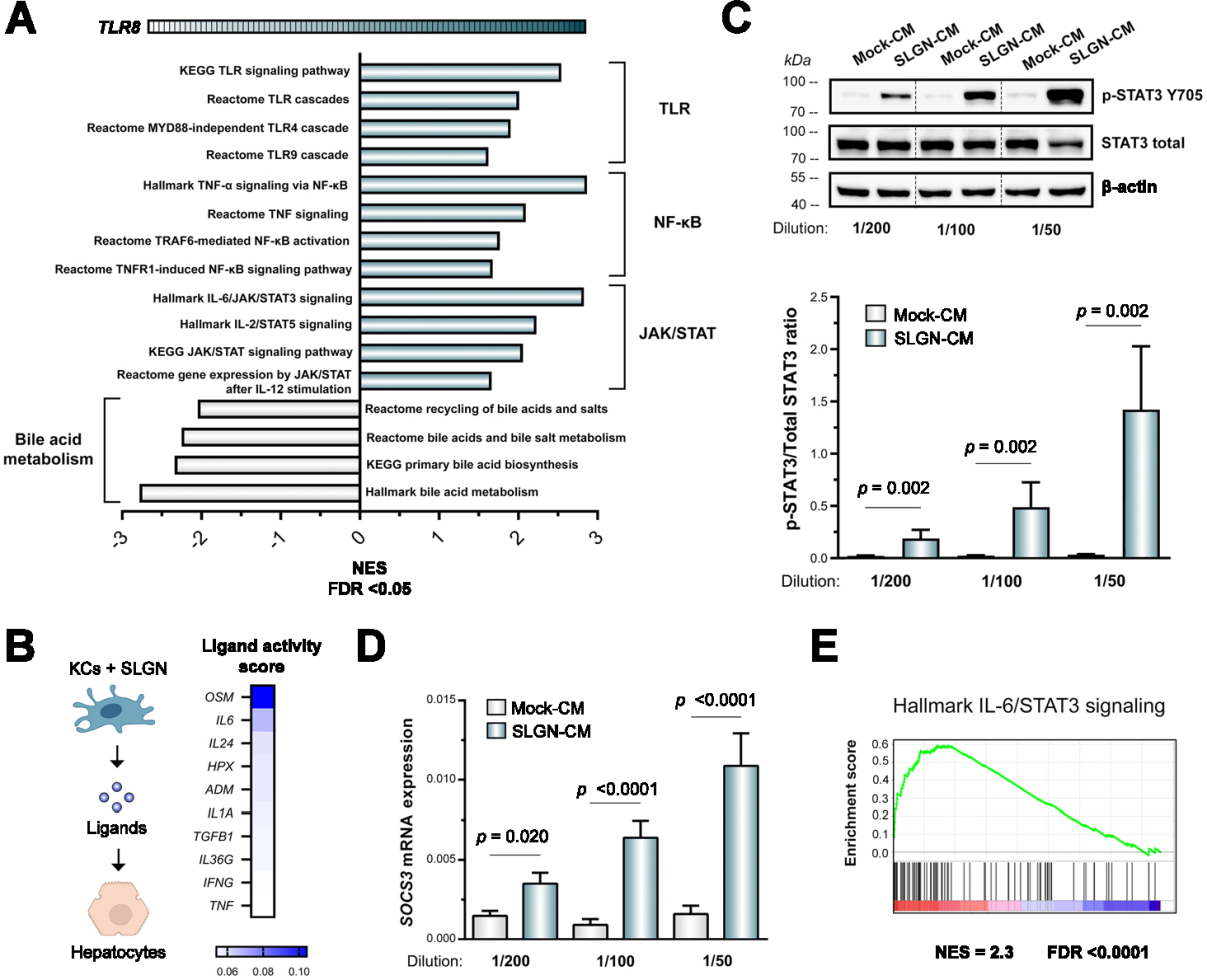
SLGN indirectly activates STAT3 signalling in hepatocytes. (**A**) GSEA of liver transcriptomic data from HBV-infected patients according to high *TLR8* mRNA expression (FDR<0.05, n=83, GSE65359). (**B**) NicheNet ligand activity scores for soluble KC factors potentially mediating the transcriptomic profile observed in PHH treated with SLGN-CM. (**C**) Treatment of PHH (15 min) with decreasing dilutions of SLGN-CM leads to an increased STAT3 Y705 phosphorylation. Quantification of band intensities for p-STAT3 and total STAT3 are presented as ratio means±SEM (Mann-Whitney test, n=3). (**D**) Treatment of PHH (72 hours) with decreasing dilutions of SLGN-CM leads to an increased *SOCS3* expression (Mann-Whitney test, n=3). Bars represent mean±SEM. (**E**) GSEA of PHH treated with SLGN-CM (1/50, 72 hours) showing a positive enrichment of the IL-6/STAT3 signalling pathway (FDR<0.0001). ADM, adrenomedullin; CM, conditioned media; GSEA, gene set enrichment analysis; HPX, haemopexin; IFNG, interferon gamma; IL, interleukin; JAK, Janus kinase; KCs, Kupffer cells; NES, normalised enrichment score; OSM, oncostatin M; SLGN, selgantolimod; SOCS3, suppressor of cytokine signalling 3; STAT3, signal transducer and activator of transcription 3; TGFB1, transforming growth factor beta 1; TNF, tumour necrosis factor; TLR8, toll-like receptor 8.

In order to explore which hepatocyte signalling pathways could potentially explain this impaired HBV entry in response to SLGN-CM, we performed GSEA according to the expression of *TLR8* using liver transcriptomic data from HBV-infected patients.^38 39^ As expected, we observed that high *TLR8* expression was associated with an increased activity of pathways implicated in TLR and NF-κB signalling, which was accompanied by a decrease of pathways regulating bile acid metabolism, suggesting the former to reflect KC activation and the latter the decrease of *NTCP* expression in hepatocytes (figure 5A, online supplemental figure S6B). In addition, JAK/STAT signalling and the IL-6/STAT3 pathway in particular were significantly associated with a high *TLR8* expression (FDR<0.001). This is consistent with the NicheNet analysis that predicted OSM and IL-6, two STAT3-activating cytokines,^42^ as the top potential ligands inducing the differentially expressed genes observed in PHH treated with SLGN-CM (figure 5B). Subsequently, we showed that treatment of PHH with SLGN-CM induced a significant and dose-dependent increase of STAT3 phosphorylation (p<0.05) (figure 5C), the upregulation of its negative regulator suppressor of cytokine signalling 3 (*SOCS3*) (p<0.05) (figure 5D) and an enhanced transcriptional activity of the STAT3 pathway as a whole (FDR<0.0001) (figure 5E). These results suggest STAT3 activation to be central in the hepatocyte transcriptomic changes in response to SLGN-CM.

### SLGN indirectly impairs HBV entry via an IL-6-dependent mechanism

**Figure 6:**
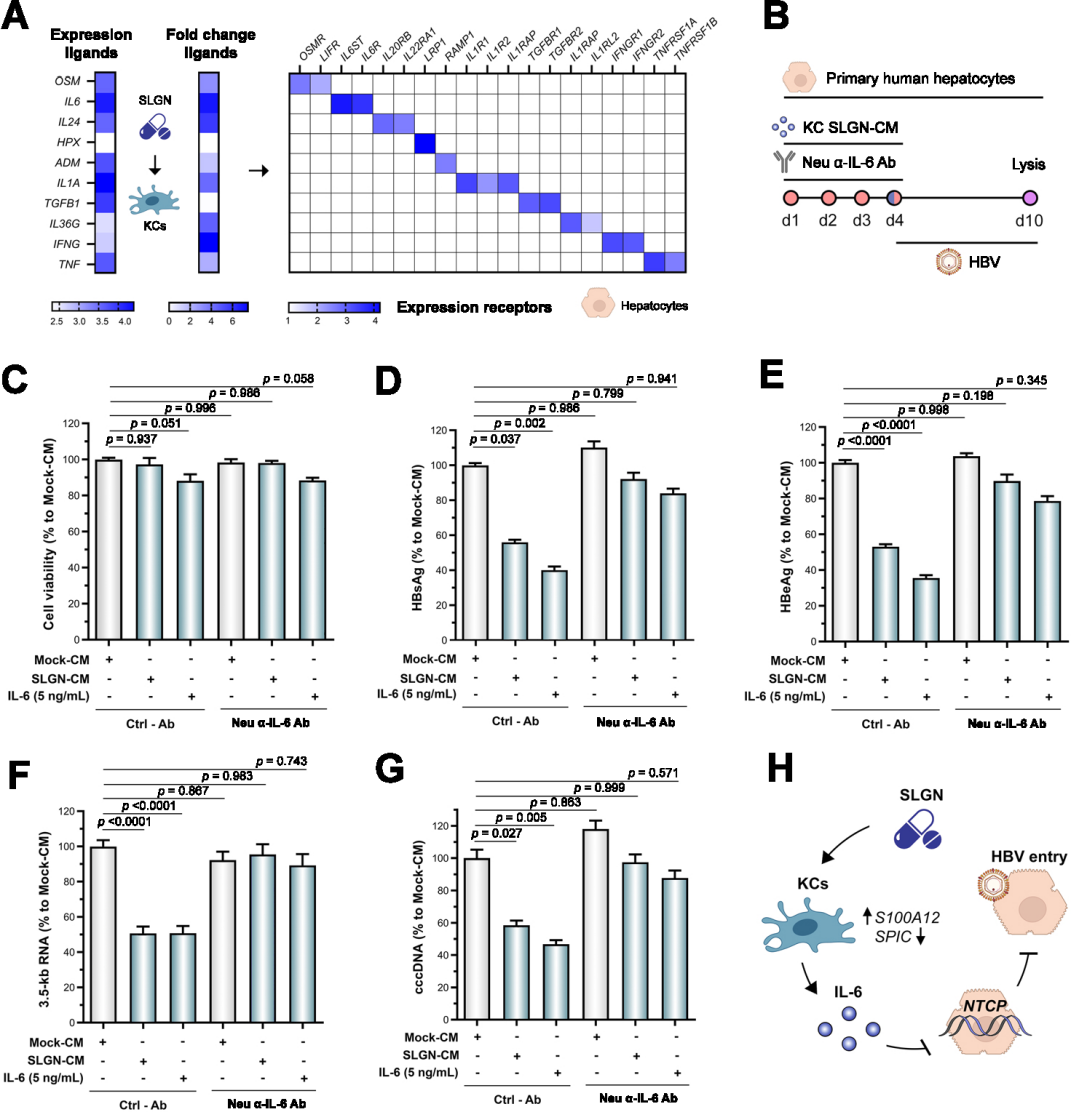
SLGN indirectly impairs HBV entry via an IL-6-dependent mechanism. (**A**) Prioritisation of KC ligands predicted by NicheNet, based on expression of KC ligands (left), fold change in response to SLGN treatment (centre) and receptor expression for these ligands in PHH (right). Expression levels represent log-transformed base means. (**B**) Experimental protocol for the treatment of PHH with SLGN-CM (1/50, 72 hours) in combination with an IL-6-neutralising antibody prior HBV inoculation (6 days). (**C-G**) Treatment of PHH with an IL-6-neutralising antibody prior HBV infection is able to prevent the decrease in HBsAg, HBeAg, 3.5 kb RNA and cccDNA levels observed with SLGN-CM alone (one-way analysis of variance, n=4). IL-6 (5 ng/mL) was used as positive control. Bars represent mean±SEM. (**H**) The TLR8 agonist SLGN regulates KC differentiation status and indirectly impairs HBV entry into hepatocytes via an IL-6-dependent mechanism. ADM, adrenomedullin; cccDNA, covalently closed circular DNA; CM, conditioned media; HBeAg, hepatitis B e antigen; HBsAg, hepatitis B surface antigen; HPX, haemopexin; IFNG, interferon gamma; IL-6, interleukin 6; KCs, Kupffer cells; NTCP, sodium/taurocholate cotransporting polypeptide; OSM, oncostatin M; PHH, primary human hepatocytes; SLGN, selgantolimod; TGFB1, transforming growth factor beta 1; TNF, tumour necrosis factor.

Based on the previously described observations, we aimed to identify the specific cytokine responsible for the downregulation of *NTCP* and the impaired HBV entry into hepatocytes following treatment with SLGN-CM. Therefore, we further prioritised the ligands predicted by NicheNet on the basis of their (1) expression in KCs, (2) fold change induced by SLGN and (3) level of their receptors in hepatocytes (figure 6A). These results were further validated by the analysis of scRNA-seq data from human liver tissues, showing higher basal expression levels of *IL6* in KCs and both its receptors in hepatocytes (ie, *IL6R* and *IL6ST*), as compared with other top ranked cytokines (ie, *OSM*, *IL24*, *IL1A*) (online supplemental figure 8). Based on these analyses showing that IL-6 had markedly higher levels across these three parameters, in addition to a previous report describing the regulatory role of IL-6 over *NTCP* expression,^43^ we selected IL-6 as the most probable ligand. Furthermore, IL-6-neutralising antibody administration in PHH exposed to SLGN-CM (figure 6B), reverted the decrease of HBV viral parameters previously observed with SLGN-CM alone (p=0.999) (figure 6C–G). These results are in line with the analysis of liver transcriptomic human data obtained in two patients with CHB at baseline and 2.5–3 hours after SLGN dosing (GS-US-389-5458), showing that in vivo SLGN treatment is associated with a decrease in *NTCP* expression and the upregulation of *IL6* and C reactive protein (*CRP*) (online supplemental figure 9). Although treatment with recombinant OSM and IL-1α at high concentrations (5 ng/mL) was able to markedly decrease *NTCP* expression in PHH and impair HBV entry (online supplemental figure 10), these cytokines did not have a significant impact at the concentrations present in the SLGN-CM (<5 pg/mL). Moreover, OSM- or IL-1α-neutralising antibodies did not revert the phenotype (online supplemental figures 11–13). Altogether, our results demonstrate that SLGN indirectly impairs HBV entry into hepatocytes via an IL-6-dependent mechanism.

## Discussion

The results of our investigation have several important implications with respect to virus-host cell interactions in the liver. The first one is that, in addition to its previously described therapeutic effect in HBV-infected hepatocytes,^10^ we show that SLGN also impairs viral entry (figures4E,F and 6CG). A similar dual mechanism has been described for other molecules currently under clinical evaluation, such as the antisense oligonucleotide bepirovirsen, which, in addition to target HBV sequences in hepatocytes, is also internalised by immune cells in the liver microenvironment and induces an inflammatory response, likely via TLR8 signalling.^44^ Altogether, based on its modulatory activity over cytokine expression and effectors of adaptive immunity, and its effect on both established and de novo HBV infection of hepatocytes, SLGN may be an interesting asset for the clinical development of combination therapies for CHB. Moreover, the downregulation of *NTCP* may be of therapeutic interest not only for HBV, but also potentially for chronic hepatitis delta virus infection.

Second, although our in vitro and in vivo results show that IL-6 is the main cytokine implicated in SLGN’s action over HBV entry, this study represents the first formal report describing an OSM-mediated impairment of HBV entry into hepatocytes (online supplemental figure 10). This is in line with previous results showing the impaired expression of *NTCP* in PHH on OSM treatment.^45^ Thus, the recent work by Ye *et al* reporting that OSM is able to inhibit HBV replication,^46^ in combination with our observations describing its role over HBV entry, further highlights OSM as a cellular factor of potential relevance for the HBV cycle.

Third, our observation that SLGN administration leads to increased levels of *PDL1* expression in KCs (figure 2B, online supplemental figure 2B) is in agreement with previous reports describing a similar effect induced by SLGN in PMN‐MDSCs.^11^ This has also been reported in chimpanzees treated with the TLR7 agonist vesatolimod.^47^ Thus, these observations further support the rationale to explore combination therapies that, in addition to TLR agonists, include checkpoint inhibitors to prevent potential repressive effects on cellular effectors of the adaptive immune system. A recent clinical trial has been initiated to evaluate a sequential therapeutic strategy, consisting on administration of the siRNA VIR-2218 to decrease circulating HBV antigen levels, followed by SLGN to induce inflammatory responses against the virus, and concluding with nivolumab to prevent the potential activation of immune checkpoints (NCT04891770). Of note, a recent phase 2 study with TLR7 agonist+anti-PD-L1 mAb treatment for 24 weeks demonstrated that this combination is generally well tolerated and was reported to have a reasonable safety profile.^48^

Fourth, our work highlights how KCs would be not just an end-state of differentiation, and that is possible to pharmacologically modulate the plasticity of this cell type. Interestingly, we observed a switch in KC morphology and transcriptomic programmes following SLGN treatment (figure 2). Moreover, our results showing that a similar gene expression profile can be induced by LPS treatment suggests this to be the consequence of general mechanisms taking place during inflammatory responses (online supplemental figure 2B). In support of this notion, the loss of KC identity has recently been reported in animal models of liver fibrosis, suggesting that this event could indeed take place in vivo.^49^ These observations have relevant biological and practical implications, as KCs undergoing a transient inflammatory response could inadvertently be labelled as populations along the monocyte-derived cell spectrum based on scRNA-seq data. Moreover, there is currently no widely accepted nomenclature to annotate hepatic monocyte/macrophage populations.^36^ Thus, our work represents a clear example that underscores the value of generating functional liver transcriptomic data at single-cell resolution. This would allow to address less descriptive but fundamental questions on liver biology, such as if we are observing two closely related cell types or one cell type in two transcriptional states.^50^ This type of question could be explored through the access of sequential samples of CHB patients using fine-needle aspirates,^51 52^ and the use of in vivo and ex vivo models for the characterisation of inflammatory responses.^53 54^

In conclusion, we show that SLGN triggers KC plasticity to promote the activation of antiviral transcriptional programmes over those associated with the KC identity. Consequently, KCs communicate with hepatocytes via IL-6, instructing them to reduce *NTCP* levels, thereby decreasing their susceptibility to HBV infection (figure 6H). These observations have relevant implications for the clinical development of novel combination therapies for CHB and the identification of intrahepatic biomarkers associated with innate immune activation.

## Supplementary material

10.1136/gutjnl-2023-331396 online supplemental file 1

## Data Availability

Data are available in a public, open access repository.
