# Supplementary material for: TLR8 agonist selgantolimod regulates Kupffer cell differentiation status and impairs HBV entry into hepatocytes via an IL-6-dependent mechanism
Source: Gut. 2024 May 2;73(12):e331396. doi: 10.1136/gutjnl-2023-331396 (PMC12210347; doi:10.1136/gutjnl-2023-331396)
Supplement: online supplemental file 1 [file gutjnl-73-12-s001.pdf]

SUPPLEMENTAL MATERIAL

The TLR8 agonist Selgantolimod regulates Kupffer cell differentiation status and impairs HBV entry into hepatocytes via an IL-6-dependent mechanism

Armando Andres Roca Suarez, Marie-Laure Plissonnier, Xavier Grand, Maud Michelet, Guillaume Giraud, Maria Saez-Palma, Anaëlle Dubois, Sarah Heintz, Audrey Diederichs, Nicolaas Van Renne, Thomas Vanwolleghem, Stephane Daffis, Li Li, Nikita Kolhatkar, Yao-Chun Hsu, Jeffrey J. Wallin, Audrey H. Lau, Simon P. Fletcher, Michel Rivoire, Massimo Levrero, Barbara Testoni, Fabien Zoulim

Table of contents:

Supplemental methods **1**
Supplemental figure S1 .... **6**
Supplemental figure S2 .... **7**
Supplemental figure S3 .... **8**
Supplemental figure S4 .... **9**
Supplemental figure S5 .... **10**
Supplemental figure S6 .... **11**
Supplemental figure S7 .... **12**
Supplemental figure S8 .... **13**
Supplemental figure S9 .... **14**
Supplemental figure S10 .... **15**
Supplemental figure S11 .... **16**
Supplemental figure S12 .... **17**
Supplemental figure S13 .... **18**
Supplemental table S1 .... **19**
Supplemental table S2 .... **20**
Supplemental references **21**

SUPPLEMENTAL METHODS:

Chromatin immunoprecipitation (ChIP)-qPCR

Primary human hepatocytes (PHH) were crosslinked for 10 min at room temperature (RT) with 1% formaldehyde (Sigma-Aldrich, St. Louis, MO, USA). Crosslinking was then quenched for 5 min with 0.125 M Glycine (Sigma-Aldrich, St. Louis, MO, USA). Crosslinked cells were lysed in lysis buffer (5 mM PIPES, 85 mM KCl, 0.5% NP-40) supplemented with 1x protease inhibitor cocktail (PIC) (Roche diagnostics, Basel, CH). Lysates were dounced 10x, centrifuged 5 min at 3000 rpm at 4 °C and resuspended in sonication buffer (50 mM HEPES pH 7.5, 140 mM NaCl, 1 mM EDTA, 1% Triton-X100, 0.1% Na-desoxycholate, 1% SDS) supplemented with 1x PIC. Chromatin was sheared for 13 cycles of 30 sec ON, 30 sec OFF on a Bioruptor (Diagenode, Liege, BE). Sheared chromatin was then diluted with RIPA buffer (10 mM Tris-HCl pH 7.5, 140 mM NaCl, 1 mM EDTA pH 8, 0.5 mM EGTA pH 8, 1% Triton-X100, 0.1% SDS, 0.1% Na-desoxycholate) supplemented with 1x PIC and incubated 2 h at 4 °C with protein G-conjugated Dynabeads (Thermo Fisher Scientific, Waltham, MA, USA). Beads were removed and a small aliquot was collected for the input control. Chromatin was incubated with 2 µg of antibodies overnight at 4 °C (**online supplemental table S1**). The mixture was further incubated 1 h at 4 °C with protein G-conjugated Dynabeads. Beads were then washed 5x with RIPA buffer supplemented with 1x PIC and once with TE buffer (10 mM Tris-HCl pH 8, 10 mM EDTA pH 8). Elution buffer (Tris-HCl pH 7.5, 5 mM EDTA pH 8, 50 mM NaCl) supplemented with 1% SDS and 50 µg/mL proteinase K (Eurobio Scientific, Les Ulis, FR) was then added and samples were decrosslinked 2 h at 68 °C. Beads were removed and one volume of Phenol:Chloroform:Isoamyl alcohol (25:24:1) was added. After a centrifugation of 15 min RT at 12700 rpm, the top phase was collected, precipitated with one volume of isopropanol and 1 µL glycogen and centrifuged 20 min at 12700 rpm 4 °C. Chromatin pellets were then washed with 70% EtOH, air-dried and resuspended in nuclease-free water. SYBR Green-based qPCR was performed as described above. Primer sequences can be found in the **online supplemental table S2**. Amplicons were subsequently loaded on 2% agarose gels containing SYBR Safe and revealed on a ChemiDoc MP Imaging System (Bio-Rad, Hercules, CA, USA). The signals were quantified using Image Lab software (v5.2.1, Bio-Rad, Hercules, CA, USA) and expressed as percentage of input (n = 3).

Immunofluorescence studies

KCs were fixed with 4% paraformaldehyde for 10 min at RT. Cells were washed with PBS 3x and permeabilized for 1 h at RT with PBS containing 0.1% Triton X-100 and 1% bovine serum albumin. Following wash, cells were incubated with primary antibodies or isotype controls in a humidified chamber overnight at 4 °C. Cells were washed 3x and incubated with secondary antibodies for 1 h at RT in the dark (**online supplemental table S1**). Cells were washed 3x and stained with DAPI for 1 min before a final wash and mounting. Images were taken with an Eclipse Ts2R-FL inverted microscope (Nikon, Minato City, Tokyo, JP) and the Imaging System Evos FL auto 2 (v2.0.2094.0, Thermo Fisher Scientific, Waltham, MA, USA).

Western blotting

Protein lysates of untreated KCs and PHH, and PHH treated with SLGN- or Mock-CM for 15 min (n = 3, in biological duplicates) were prepared with lysis buffer 6 (R&D Systems, Minneapolis, MN, USA) supplemented with PIC (Roche diagnostics, Basel, CH) and phosphatase inhibitor cocktails 2 and 3 (Sigma-Aldrich, St. Louis, MO, USA). Samples (30 µg) were loaded in 12% polyacrylamide gels and migrated at 200 V for 40 min, followed by transfer to nitrocellulose membranes (Bio-Rad, Hercules, CA, USA). Membranes were incubated with primary antibodies, followed by PBS wash 3x and incubation with secondary antibodies (**online supplemental table S1**). Blots were revealed using a ChemiDoc MP Imaging System (Bio-Rad, Hercules, CA, USA). Band signals were quantified using the Image Lab software and expressed as phosphorylated signal transducer and activator of transcription 3 (p-STAT3)/Total STAT3 ratio.

Nucleic acid extraction and RT-qPCR

Lysates from KCs treated with SLGN (150 nM, 24 h, n = 4) or PHH treated with SLGN-CM (1/50) and recombinant cytokines (4 - 72 h, n = 3) were processed using the MasterPure DNA and RNA purification kit (Lucigen, Middleton, WI, USA) according to the manufacturer's instructions. RNA samples (500 ng) were reverse-transcribed with the SuperScript IV Vilo Master Mix and qPCR was performed using SYBR Green Master Mix (Applied Biosystems, Waltham, MA, USA). Samples from HBV-infected PHH were processed according to the ICE-HBV guidelines,[1] using TaqMan Master Mix (Applied Biosystems, Waltham, MA, USA). Hemoglobin subunit beta (*HBB*) and glucuronidase beta (*GUSB*) served as internal reference for HBV covalently closed circular (ccc)DNA and 3.5-kb RNA quantification, respectively. All

reactions were carried with the QuantStudio 7 Flex System (Thermo Fisher Scientific, Waltham, MA, USA). Primer sequences can be found in the **online supplemental table S2**.

Detection of HBV antigens

HBsAg and HBeAg were detected in PHH and HepaRG supernatants at day 6 post-infection by ELISA, using the chemiluminescence immunoassay kit (Autobio Diagnostic, Zhengzhou, Henan, CN), according to the manufacturer's instructions.

Generation of monocyte-derived macrophages

Peripheral blood mononuclear cells (PBMCs) from healthy blood donors were isolated by Ficoll gradient, as previously described.[2] Monocytes were isolated from total PBMCs by negative selection with the Pan Monocyte Isolation kit (Miltenyi Biotec, Bergisch Gladbach, DE). Monocytes were seeded at $1.4x10^6$ cells/well into 6-well plates and exposed to 50 ng/mL of granulocyte-macrophage colony-stimulating factor (GM-CSF) or macrophage colony-stimulating factor (M-CSF) (Miltenyi Biotec, Bergisch Gladbach, DE) during six days in order to generate monocyte-derived macrophages (MDMs).

Animal experiments

The animal protocol and all procedures involving cynomolgus monkeys (*Macaca fascicularis*) were reviewed and approved by the Covance IACUC and adhered to the national guidelines of the Animal Welfare Act, the Guide for the Care and Use of Laboratory Animals, and the Office of Laboratory Animal Welfare. The small molecule tool agonist of TLR8 was manufactured by Gilead Sciences, Inc. (Foster City, CA). Dose formulation was prepared by Covance Laboratories (Madison, WI) and stored protected from light at RT. Individual doses were calculated based on body weight recorded on the day of administration. Male and female cynomolgus macaques were individually housed at Covance Laboratories (Madison, WI) in stainless steel cages and fed with Certified Primate Diet #2055C (Harlan Laboratories, Inc., Indianapolis, IN). Animals (n = 3/sex/group) were dosed via oral gavage on day 1, 8, 15, 22 and 29 with either vehicle [30% (w/v) polyethylene glycol 300 (PEG 300) and 70% (w/v) reverse osmosis water (pH adjusted to 3.0 ± 0.1)], or 0.1, 0.5 or 2.5 mg/kg/dose TLR8 agonist diluted in vehicle at a dose volume of 2 mL/kg. The animals were anesthetized with sodium pentobarbital and euthanized via exsanguination at approximately 4 h (± 30 min) following dosing on day 29. Liver tissue (≥10 mg) was obtained from the left lateral lobe at terminal sacrifice and flash frozen in liquid nitrogen. Isolation of total cellular RNA and RNA-seq was conducted by Expression Analysis (Durham, NC) as described previously.[3]

Human liver biopsy collection and analysis

Core needle biopsies were collected from two inactive carrier (IC) chronic HBV (CHB) patients from the clinical study GS-US-389-5458. Subjects were dosed orally with 3 mg of SLGN weekly over the course of 23 weeks. Liver biopsies were collected in RNAlater at both the time of screening and 2.5-3 h after dosing at week 23. RNA was isolated from the tissue samples and bulk RNA-seq was performed using Illumina TruSeq Kits. RNA-seq alignment was performed using TopHat and Cufflinks.[4]

Bioinformatics analyses

Bulk liver transcriptomic data from HBV-infected patients was obtained from the Gene Expression Omnibus (GEO) accessions GSE65359 (n = 83), GSE84044 (n = 118) and GSE230397 (n = 78). Data was pre-processed with the CollapseDataset tool available at GenePattern, according to max value. Pathways associated with a high *TLR8* expression in GSE65359 and GSE84044 were identified by GSEA using *TLR8* as a continuous class label. Expression levels of the SLGN-UP KC signature in GSE230397 were estimated using single-sample GSEA (ssGSEA, v10.1).

For the analysis of GSE136103,[5] only data of healthy liver samples were selected and used for downstream data integration. Briefly, raw UMI readcount matrices were processed using the Seurat toolbox (v4.9).[6] The following cell filters were employed for each sample: cells with small library sizes (<1500 counts per cell or <200 unique genes per cell) were removed. Cells with more than 10% mitochondrial transcripts were omitted. Genes that were expressed in less than three cells in a sample were omitted from that sample. Count data were normalized per cell, transformed to log scale and multiplied by a scale factor of 10,000. Highly variable features were obtained by calling Seurat's *FindVariableFeatures* function with 2000 features, after which the data was scaled using the *ScaleData* function. Dimensionality reduction was performed with the *RunPCA* function on the first 30 dimensions. The resulting gene expression matrices were integrated using Harmony into a single dataset,[7] and a dimensionality reduction plot was created by *RunUMAP* using the first 30 dimensions. Cell clusters were annotated according to canonical gene markers.

Liver single-cell (sc)RNA-seq data from cynomolgus macaques was obtained from https://db.cngb.org/nhpca/download.[8] Expression levels of the SLGN-DOWN KC signature were estimated using gene set variation analysis (GSVA, v1.44.5).[9] Graphs were generated using the *VlnPlot* and *DotPlot* functions of Seurat.

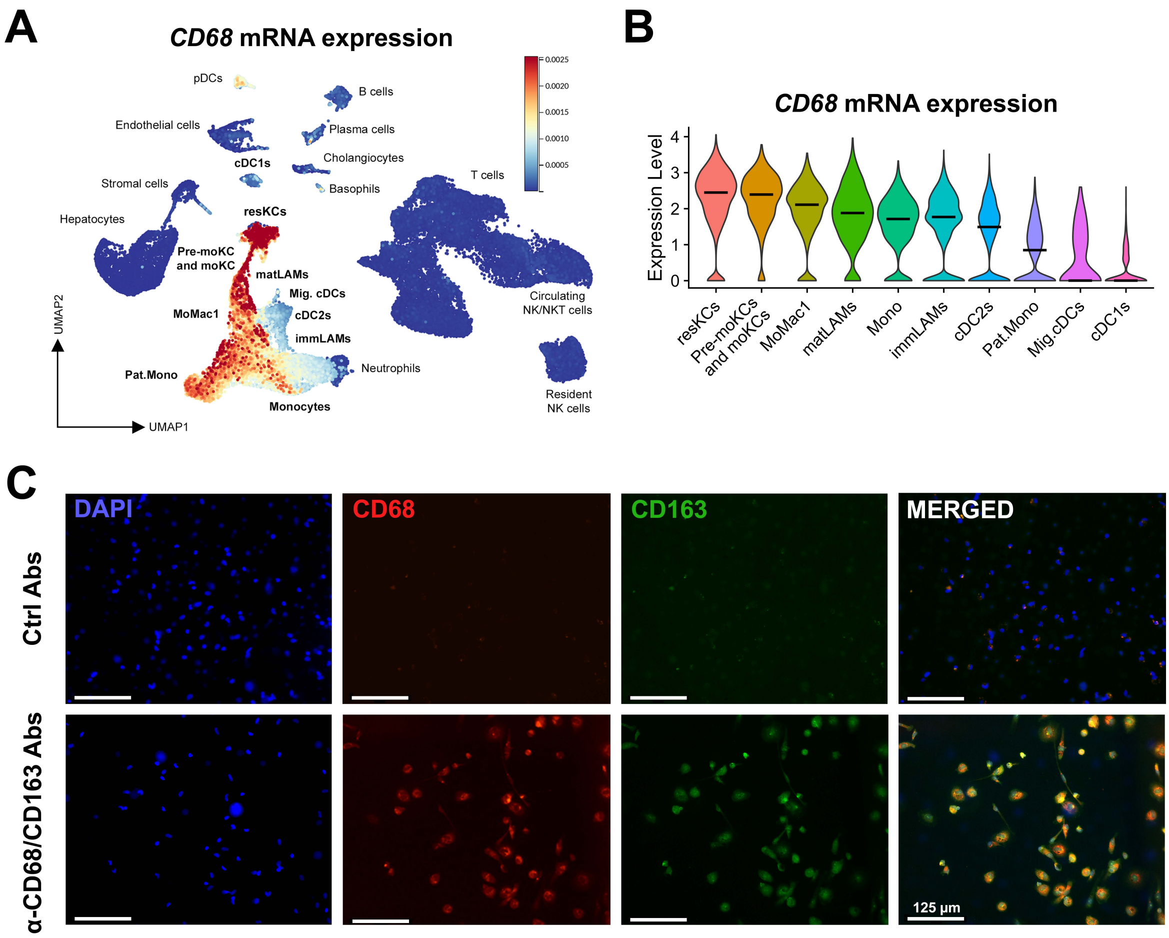

Supplemental figure S1. CD163-positive selection allows the isolation of human KCs from liver resections. (**A-B**) Expression of *CD68* mRNA in each cell population of the human liver microenvironment (GSE192742) (A) and the myeloid compartment (B). Violin plots represent mean expression values. (**C**) Immunofluorescence microscopic image of KCs, showing positive staining for nuclear DNA (DAPI, blue), CD68 (red) and CD163 (green). cDCs, classical dendritic cells; Mig.cDCs, migratory cDCs; moKCs, monocyte-derived KCs; NK cells, natural killer cells; LAMs, lipid-associated macrophages; Pat.Mono, patrolling monocytes; pDCs, plasmacytoid dendritic cells; resKCs, resident Kupffer cells.

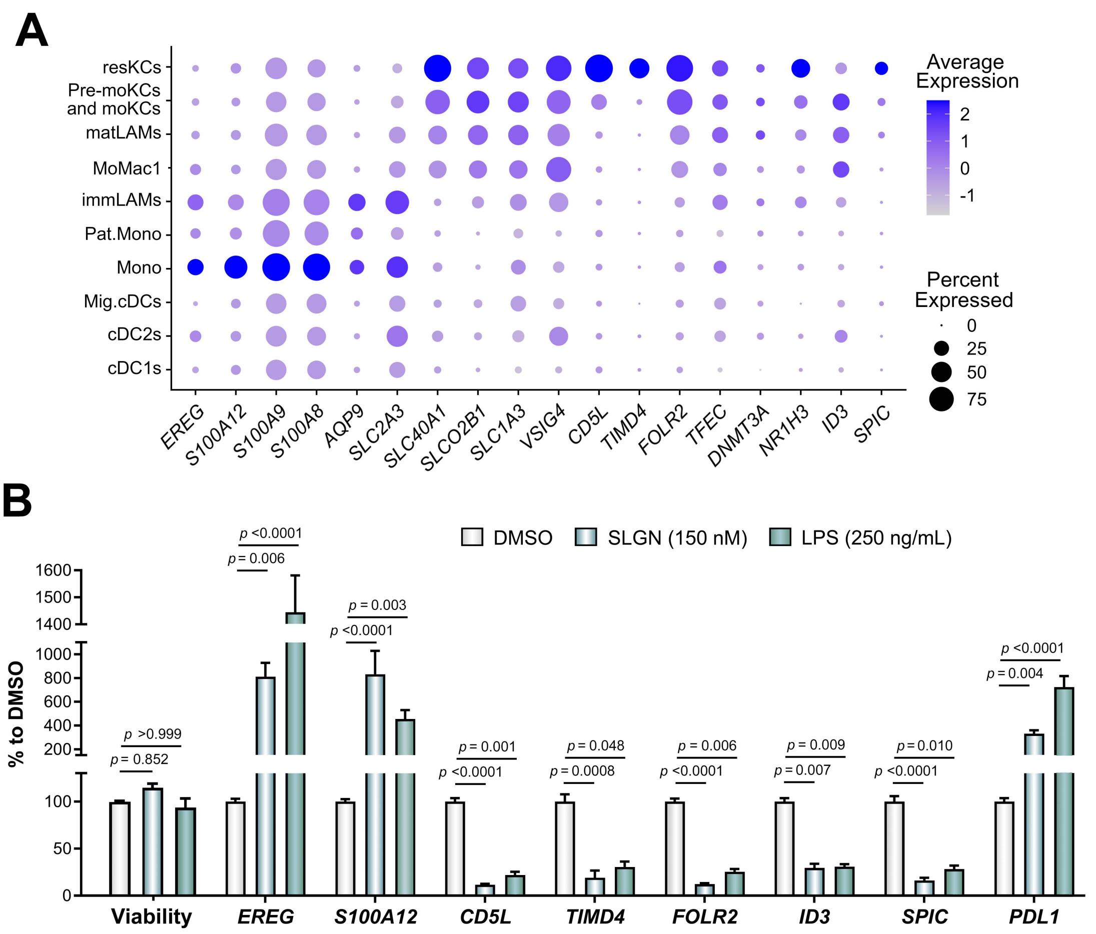

Supplemental figure S2. SLGN treatment leads to gene expression changes associated with KC differentiation status. (**A**) Dotplot showing the expression of monocyte and KC markers in each population of the hepatic myeloid compartment (GSE192742). (**B**) Treatment of KCs with SLGN (150 nM) or LPS (250 ng/mL) for 24 h induces upregulation of monocyte markers (*EREG* and *S100A12*), the downregulation of KC markers (*CD5L*, *TIMD4*, *FOLR2*, *NR1H3*, *ID3* and *SPIC*) and an increase in *PDL1* expression, as assessed by qPCR. Bars represent means ± SEM. Kruskal-Wallis test, n = 4. AQP9, aquaporin 9; CD5L, CD5 molecule like; cDCs, classical dendritic cells; DNMT3A, DNA methyltransferase 3 alpha; EREG, epiregulin; FOLR2, folate receptor beta; ID3, inhibitor of DNA binding 3; Mig.cDCs, migratory cDCs; moKCs, monocyte-derived KCs; NR1H3, nuclear receptor subfamily 1 group H member 3; LAMs, lipid-associated macrophages; LPS, lipopolysaccharides; Pat.Mono, patrolling monocytes; PDL1, programmed cell death 1 ligand 1; resKCs, resident Kupffer cells; S100A, S100 calcium binding protein A; SLC, solute carrier family; SLGN, Selgantolimod; SPIC, Spi-C transcription factor; TFEC, transcription factor EC; TIMD4, T cell immunoglobulin and mucin domain containing 4; VSIG4, V-set and immunoglobulin domain containing 4.

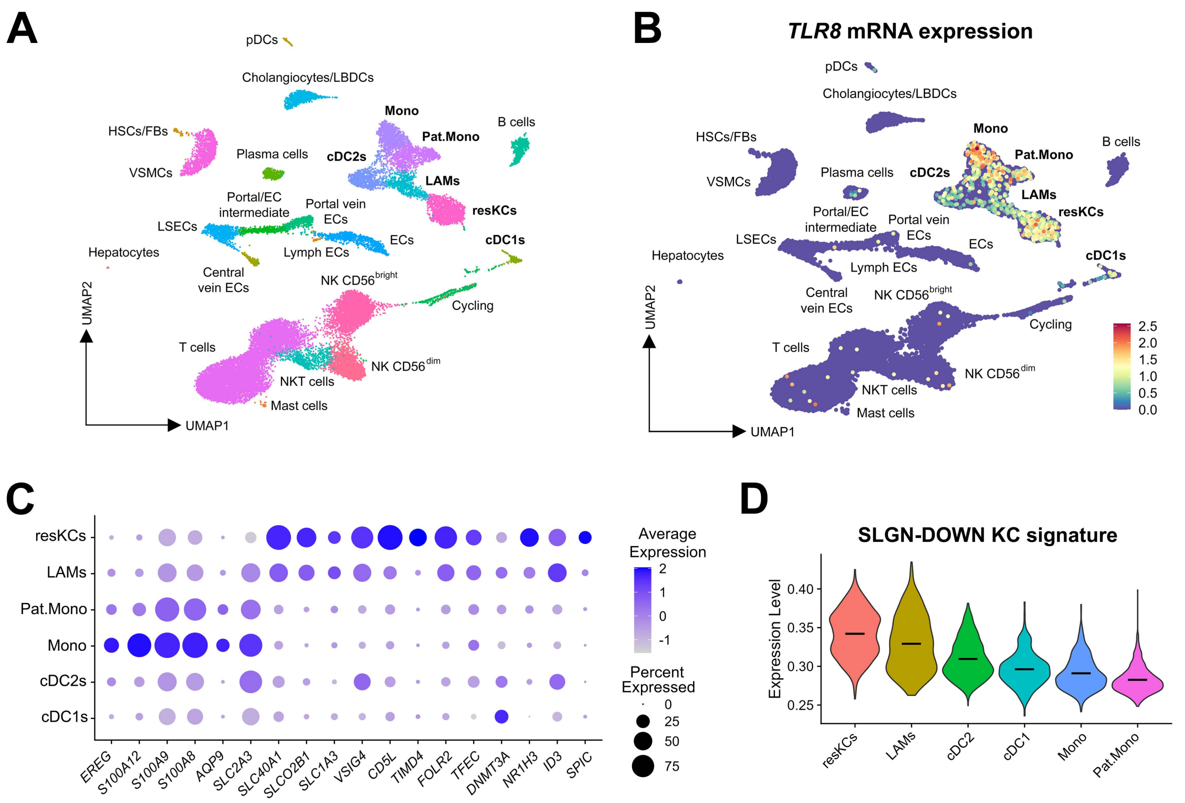

Supplemental figure S3. Validation of the KC transcriptomic profile induced by SLGN treatment. (**A**) UMAP plot depicting the major cell types identified following analysis of liver scRNA-seq data from five healthy donors (GSE136103). (**B**) Expression of *TLR8* mRNA in each cell population of the human liver microenvironment. (**C**) Dotplot showing the expression of monocyte and KC markers in each population of the hepatic myeloid compartment. (**D**) Expression levels of the SLGN-DOWN KC signature in each population of the hepatic myeloid compartment. Violin plots represent mean expression values. AQP9, aquaporin 9; CD5L, CD5 molecule like; cDCs, classical dendritic cells; DNMT3A, DNA methyltransferase 3 alpha; EREG, epiregulin; FBs, fibroblasts; FOLR2, folate receptor beta; HSCs, hepatic stellate cells; ID3, inhibitor of DNA binding 3; NR1H3, nuclear receptor subfamily 1 group H member 3; LAMs, lipid-associated macrophages; LBDCs, liver bile duct cells; LSECs, liver endothelial sinusoidal cells; Pat.Mono, patrolling monocytes; resKCs, resident Kupffer cells; S100A, S100 calcium binding protein A; SLC, solute carrier family; SLGN, Selgantolimod; SPIC, Spi-C transcription factor; TFEC, transcription factor EC; TIMD4, T cell immunoglobulin and mucin domain containing 4; VSIG4, V-set and immunoglobulin domain containing 4; VSMCs, vascular smooth muscle cells.

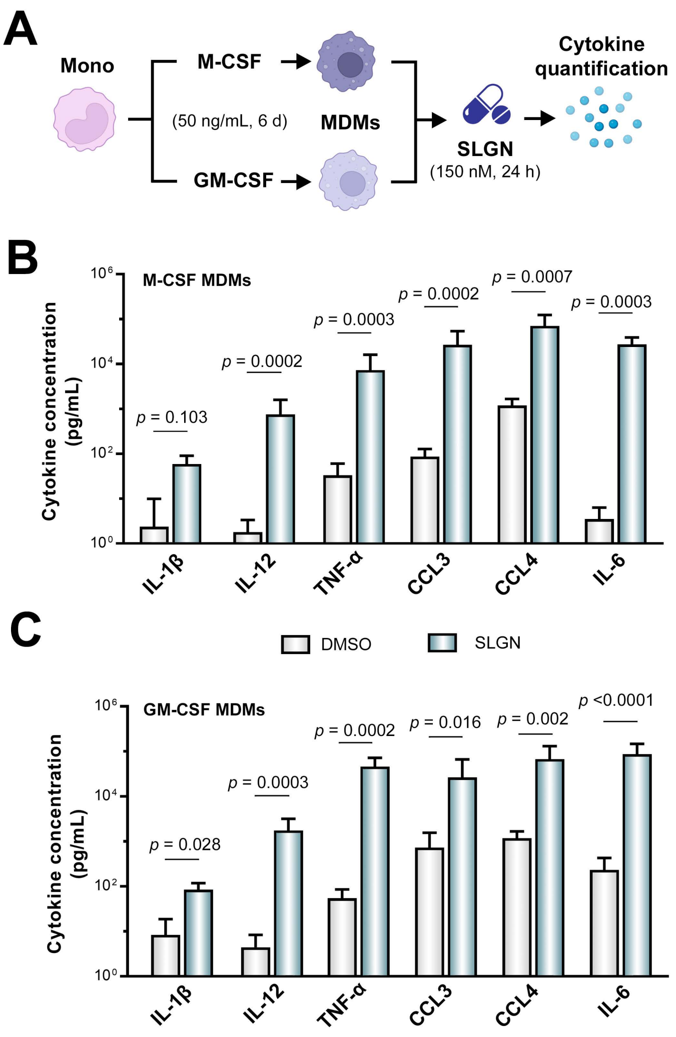

Supplemental figure S4. SLGN treatment leads to the activation of MDMs. (**A**) Method for the differentiation of monocytes into macrophages by M-CSF or GM-CSF treatment (50 ng/mL, 6 d), followed by SLGN treatment (150 nM, 24 h) and cytokine quantification. (**B**) Cytokine profile of M-CSF-differentiated macrophages treated with SLGN. (**C**) Cytokine profile of GM-CSF-differentiated macrophages treated with SLGN. Bars represent mean ± SEM. Mann-Whitney test, n = 3. CCL, C-C motif chemokine ligand; GM-CSF, granulocyte-macrophage colony-stimulating factor; IL, interleukin; M-CSF, macrophage colony-stimulating factor; MDMs, monocyte-derived macrophages; SLGN, Selgantolimod; TNF-α, tumor necrosis factor alpha.

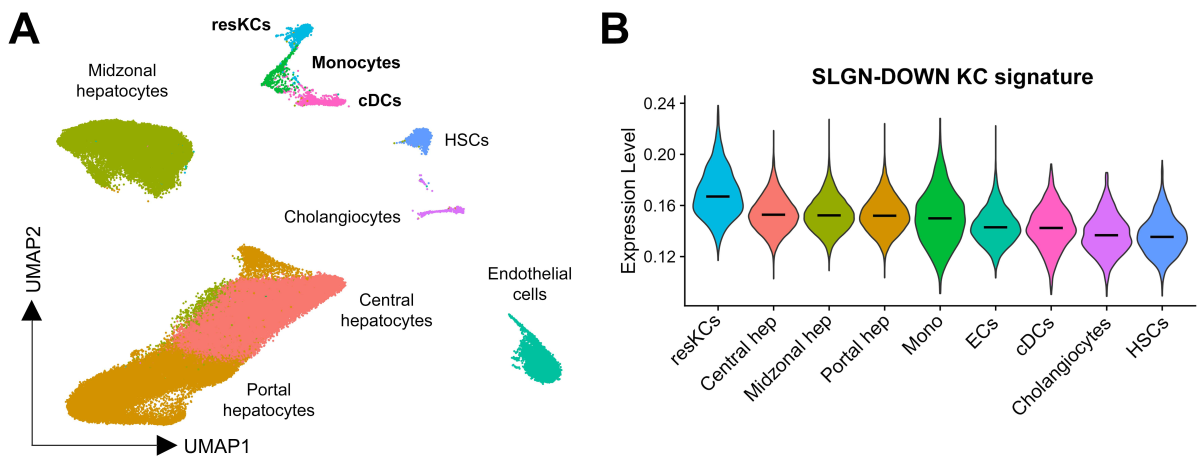

Supplemental figure S5. Expression of the SLGN-DOWN KC signature in the cynomolgus macaque liver. (**A**) UMAP plot depicting the major cell types identified following analysis of liver scRNA-seq data from cynomolgus macaques.[8] (**B**) Expression levels of the SLGN-DOWN KC signature in each cell population of the cynomolgus macaque liver. Violin plots represent mean expression values. cDCs, classical dendritic cells; HSCs, hepatic stellate cells; ECs, endothelial cells; resKCs, resident Kupffer cells; SLGN, Selgantolimod.

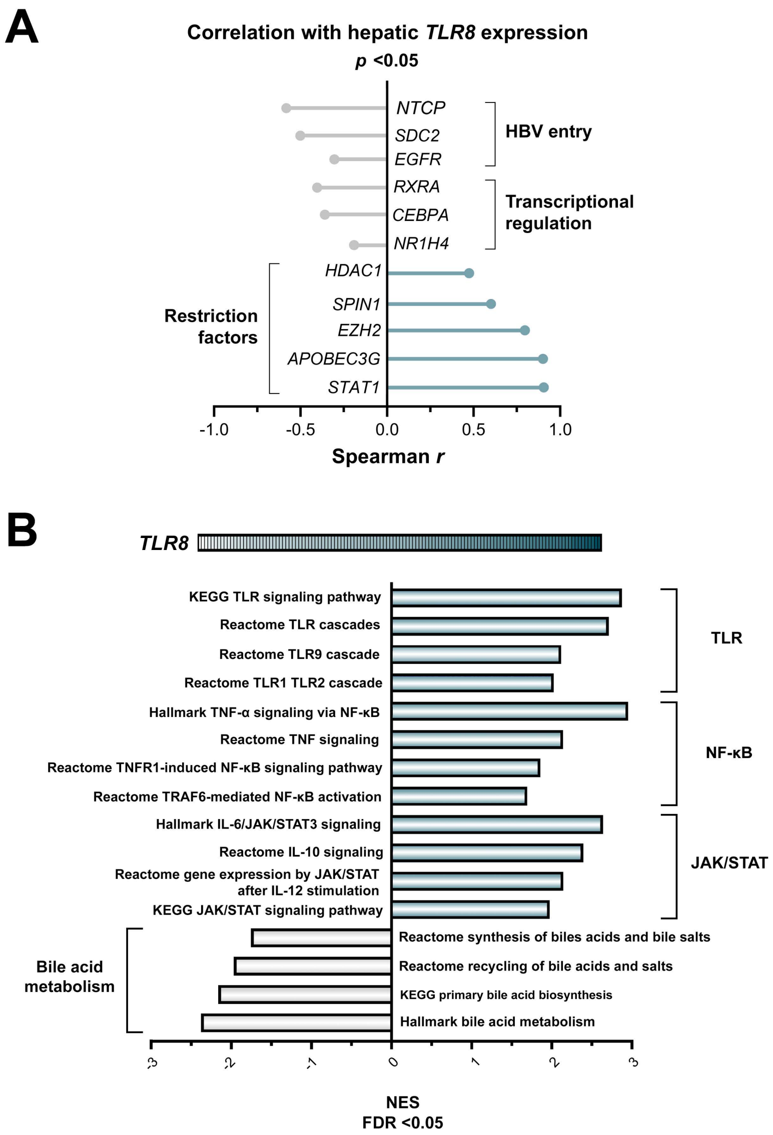

Supplemental figure S6. High hepatic *TLR8* levels are associated with low *NTCP* expression and activation of the IL-6/STAT3 signaling pathway. (**A**) Liver transcriptomic data from HBV-infected patients showing a correlation of *TLR8* expression with genes implicated in the HBV cycle (p <0.05, Spearman correlation, n = 118, GSE84044). (**B**) GSEA of liver transcriptomic data from HBV-infected patients according to high *TLR8* mRNA expression (FDR <0.05, n = 118, GSE84044). APOBEC3G, apolipoprotein B mRNA editing enzyme catalytic subunit 3G; CEBPA, CCAAT enhancer binding protein alpha; EGFR, epidermal growth factor receptor; EZH2, enhancer of zeste 2 polycomb repressive complex 2 subunit; HDAC1, histone deacetylase 1; JAK/STAT, Janus kinase/signal transducer and activator of transcription; NES, normalized enrichment score; NR1H3, nuclear receptor subfamily 1 group H member 3; NTCP, sodium/taurocholate cotransporting polypeptide; RXRA, retinoid X receptor alpha; SDC2, syndecan 2; SPIN1, spindlin 1; TNF-α, tumor necrosis factor alpha; TLR8, Toll-like receptor 8.

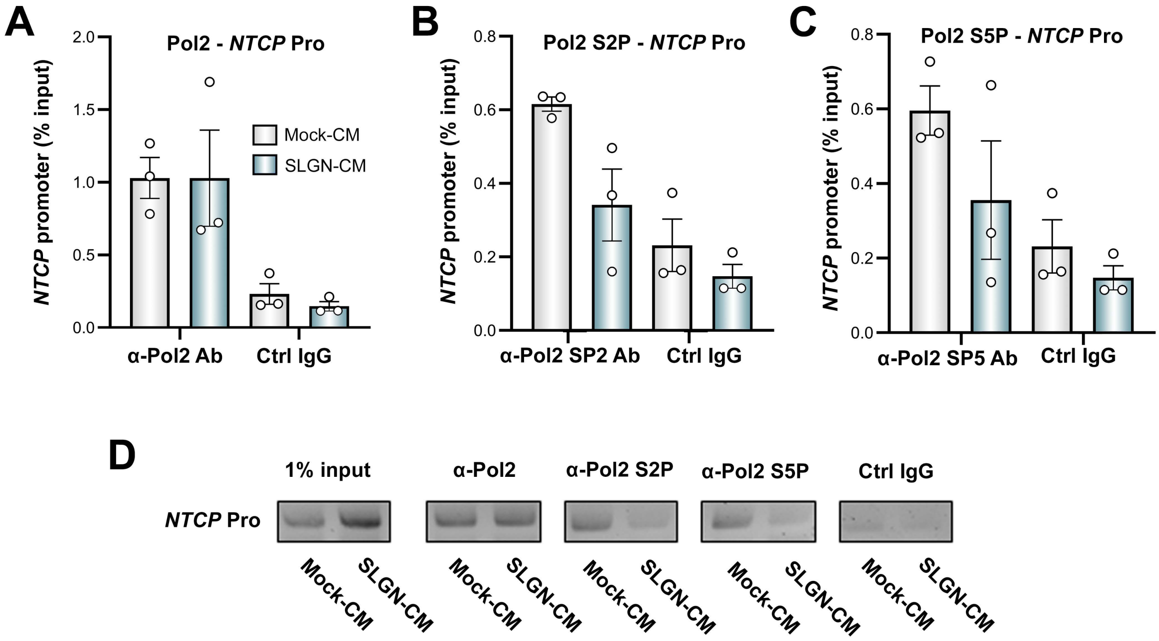

Supplemental figure S7. Impairment of *NTCP* expression takes place at the transcriptional level. PHH were cultured with SLGN- or Mock-CM (72 h) derived from KCs. ChIP-qPCR experiments were then carried in order to quantify RNA Pol2 binding to the *NTCP* promoter using antibodies against (**A**) total RNA Pol2, (**B**) S2-phosphorylated RNA Pol2 and (**C**) S5-phosphorylated RNA Pol2. Isotype IgG antibodies were used as control. Bars represent means of the input percentage ± SEM (n = 3). (**D**) Gel electrophoresis showing qPCR amplicons corresponding to the *NTCP* promoter. CM, conditioned media; NTCP, sodium/taurocholate cotransporting polypeptide; Pol2, RNA polymerase 2; SLGN, Selgantolimod.

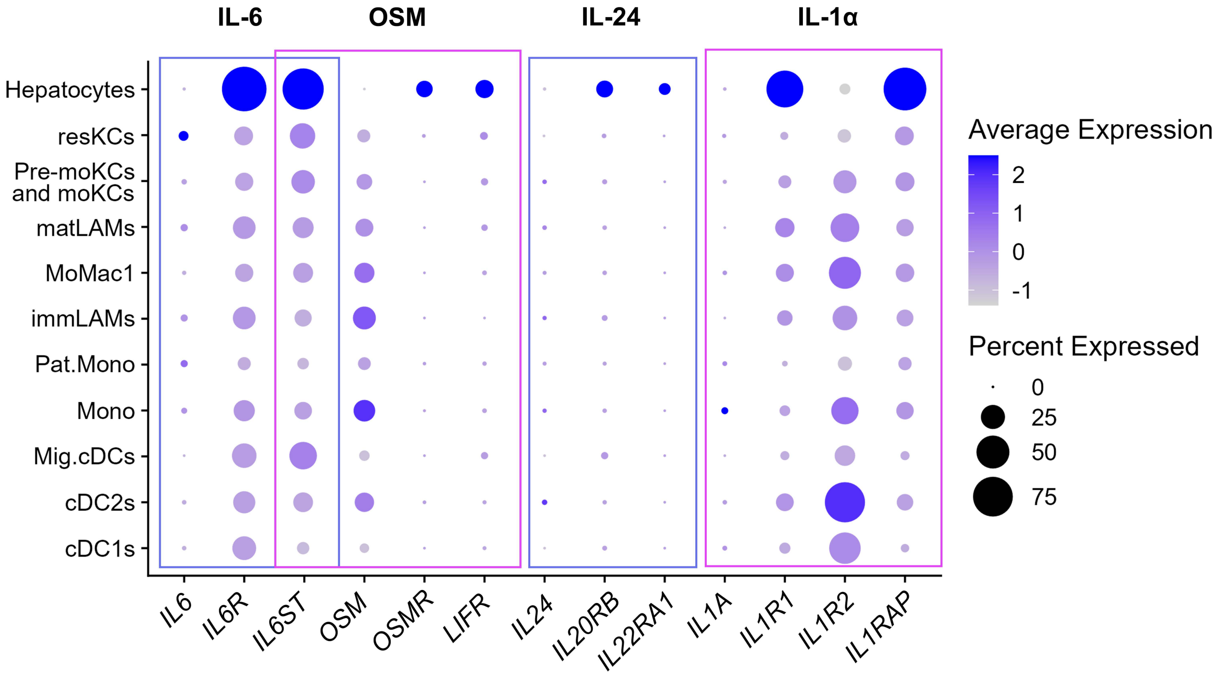

Supplemental figure S8. Expression levels of *IL6*, *OSM*, *IL24*, *IL1A* and their receptors in the human liver microenvironment. Expression of *IL6*, *OSM*, *IL24* and *IL1A* in the hepatic myeloid compartment and their receptors in hepatocytes. Human liver scRNA-seq data obtained from GSE192742. cDCs, classical dendritic cells; IL, interleukin; IL1R, interleukin 1 receptor; IL1RAP, interleukin 1 receptor accessory protein; IL6R, interleukin 6 receptor; IL6ST, interleukin 6 cytokine family signal transducer; IL20RB, interleukin 20 receptor subunit beta; IL22RA1, interleukin 22 receptor subunit alpha 1; resKCs, resident Kupffer cells; LAMs, lipid-associated macrophages; LIFR, LIF receptor subunit alpha; OSM, oncostatin M; OSMR, oncostatin M receptor.

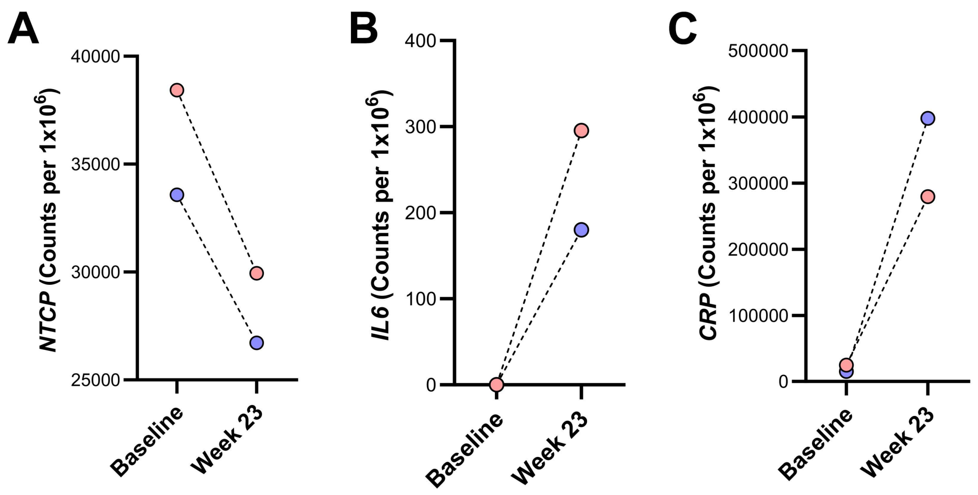

Supplemental figure S9. SLGN modulates the expression of *NTCP*, *IL6* and *CRP* in the human liver. (**A-C**) Expression of (A) *NTCP*, (B) *IL6* and (C) *CRP* mRNA in the human liver at baseline and 23 weeks post-SLGN treatment (n = 2). CRP, C reactive protein; IL6, interleukin 6; NTCP, sodium/taurocholate cotransporting polypeptide.

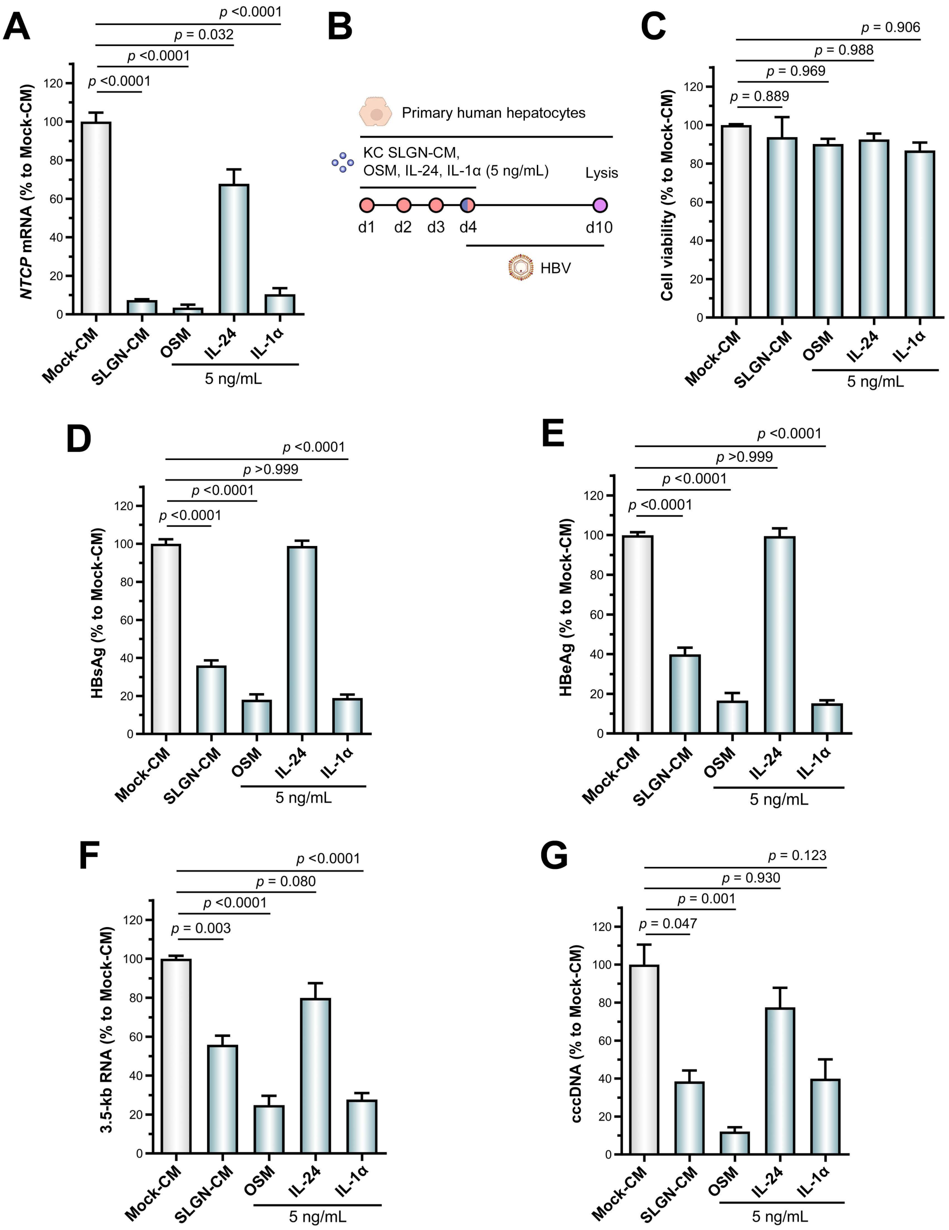

Supplemental figure S10. Recombinant OSM and IL-1α at high concentrations impair HBV entry into hepatocytes. (**A**) Treatment of PHH with SLGN-CM (1/50) or high concentrations (5 ng/mL) of OSM, IL-24 and IL-1α for 72 h, followed by *NTCP* mRNA quantification (One-way ANOVA, n = 3). (**B**) Experimental protocol for the treatment of PHH with SLGN-CM or high cytokine concentrations for 72 h prior HBV inoculation (6 d). (**C-G**) Treatment of PHH with SLGN-CM, OSM or IL-1α prior HBV infection is associated with a decrease in HBsAg, HBeAg, 3.5-kb RNA and cccDNA levels (One-way ANOVA, n = 4). Bars represent mean ± SEM. cccDNA, covalently closed circular DNA; CM, conditioned media; HBeAg, hepatitis B e antigen; HBsAg, hepatitis B surface antigen; HBV, hepatitis B virus; IL, interleukin; KCs, Kupffer cells; OSM, oncostatin M; PHH, primary human hepatocytes; SLGN, Selgantolimod.

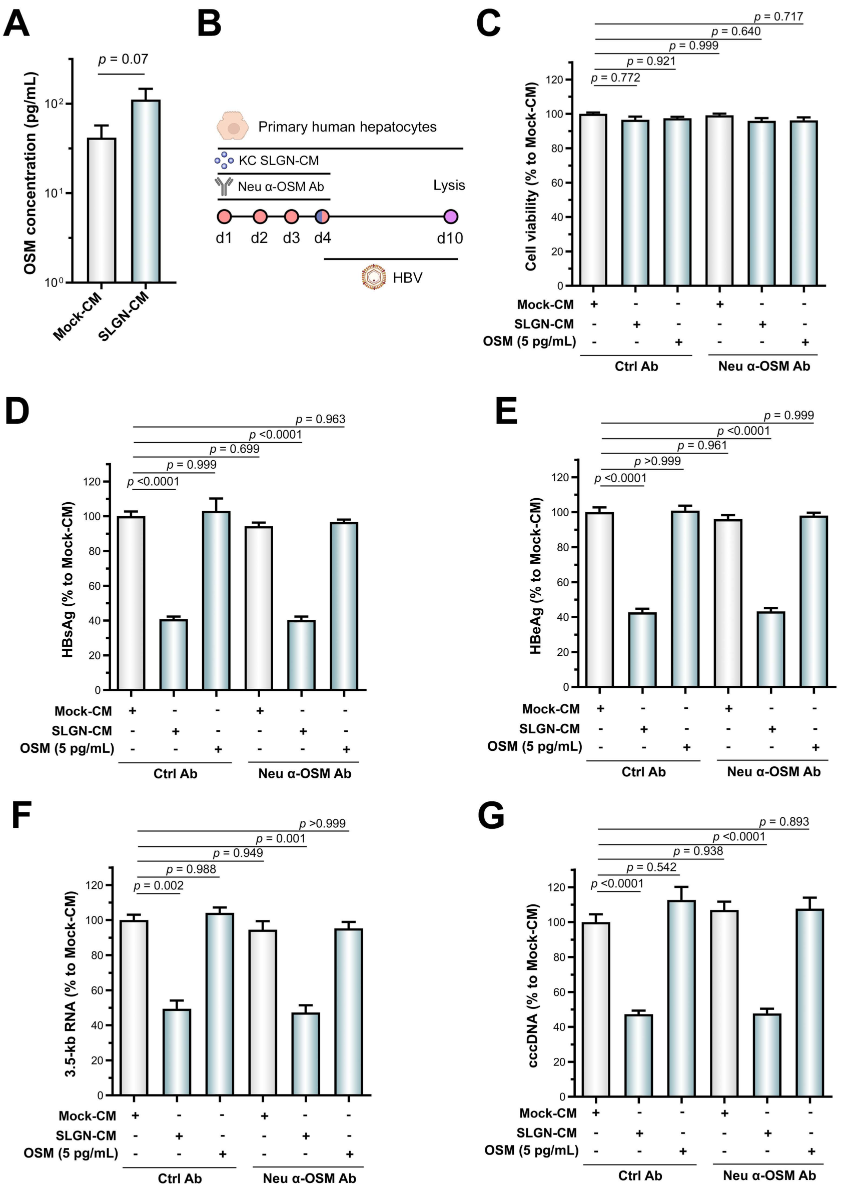

Supplemental figure S11. Neutralization of OSM in the SLGN-CM does not impact HBV entry into hepatocytes. (**A**) Quantification of OSM (n = 5) present in the KC SLGN-CM (Mann-Whitney test). (**B**) Experimental protocol for the treatment of PHH with SLGN-CM (1/50) or OSM at concentrations present in the SLGN-CM (<5 pg/mL after 1/50 dilution) in combination with OSM-neutralizing antibody for 72 h prior HBV inoculation (6 d). (**C-G**) Treatment of PHH with an OSM-neutralizing antibody prior HBV infection does not prevent the decrease in HBsAg, HBeAg, 3.5-kb RNA and cccDNA levels observed with SLGN-CM alone (One-way ANOVA, n = 3). Bars represent mean ± SEM. cccDNA, covalently closed circular DNA; CM, conditioned media; HBeAg, hepatitis B e antigen; HBsAg, hepatitis B surface antigen; HBV, hepatitis B virus; KCs, Kupffer cells; OSM, oncostatin M; PHH, primary human hepatocytes; SLGN, Selgantolimod.

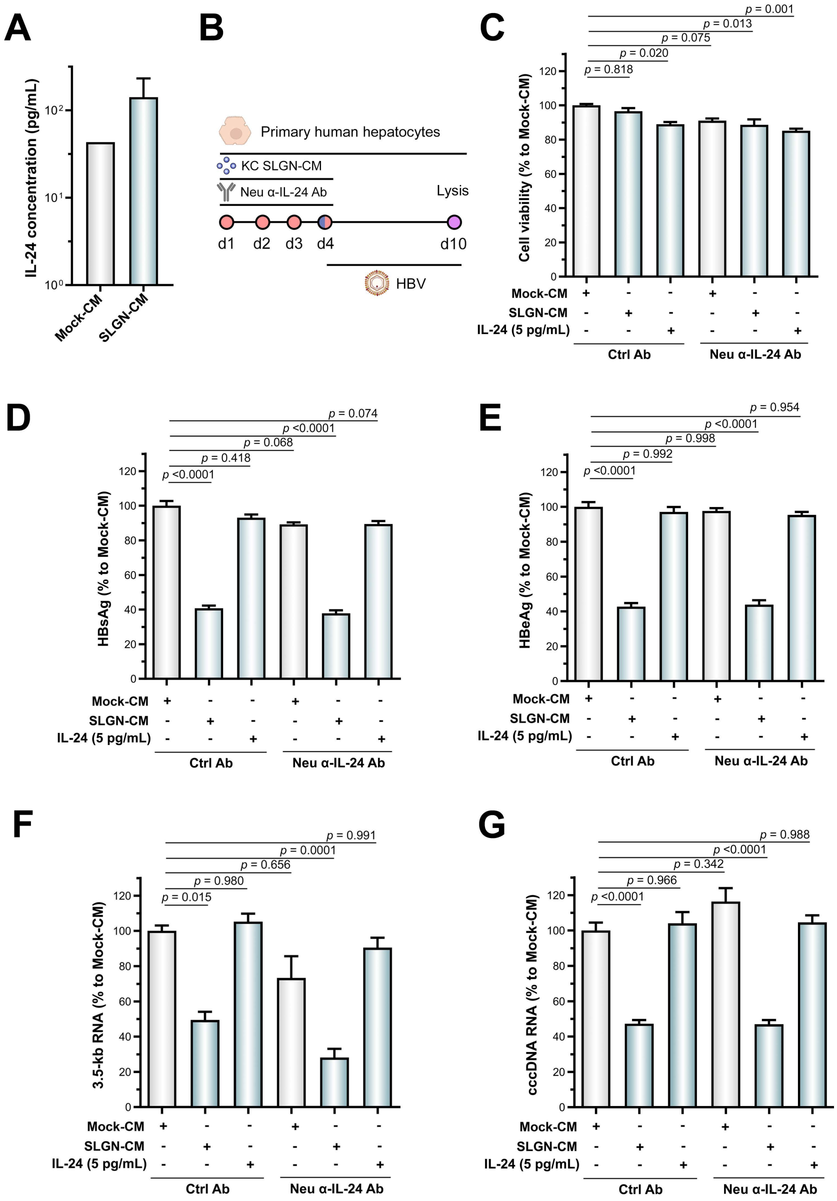

Supplemental figure S12. Neutralization of IL-24 in the SLGN-CM does not impact HBV entry into hepatocytes. (**A**) Quantification of IL-24 (n = 6) present in the KC SLGN-CM. For IL-24, 5/6 samples were under the limit of detection in the mock-CM (<62.5 pg/mL). (**B**) Experimental protocol for the treatment of PHH with SLGN-CM (1/50) or IL-24 at concentrations present in the SLGN-CM (<5 pg/mL after 1/50 dilution) in combination with IL-24-neutralizing antibody for 72 h prior HBV inoculation (6 d). (**C-G**) Treatment of PHH with an IL-24-neutralizing antibody prior HBV infection does not prevent the decrease in HBsAg, HBeAg, 3.5-kb RNA and cccDNA levels observed with SLGN-CM alone (One-way ANOVA, n = 3). Bars represent mean ± SEM. cccDNA, covalently closed circular DNA; CM, conditioned media; HBeAg, hepatitis B e antigen; HBsAg, hepatitis B surface antigen; HBV, hepatitis B virus; IL, interleukin; KCs, Kupffer cells; PHH, primary human hepatocytes; SLGN, Selgantolimod.

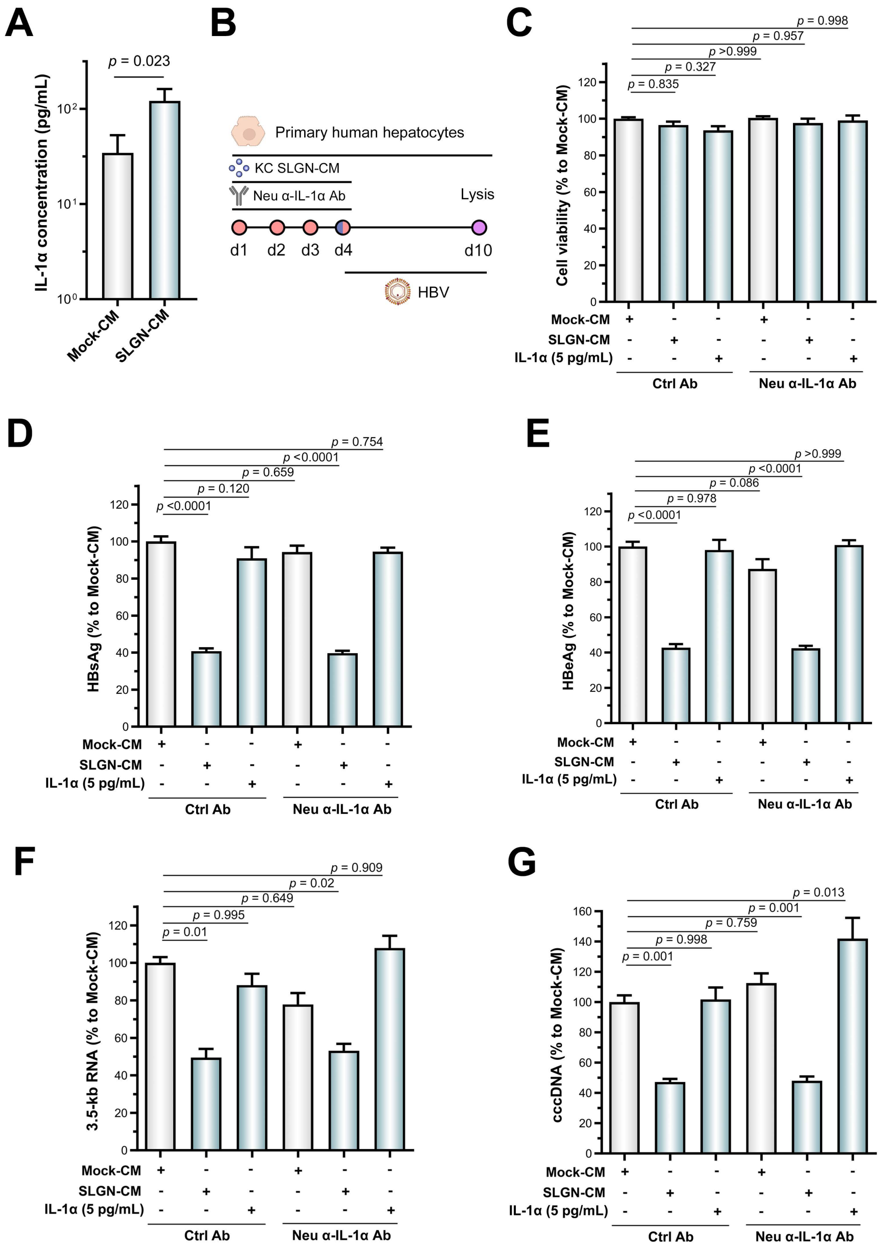

Supplemental figure S13. Neutralization of IL-1α in the SLGN-CM does not impact HBV entry into hepatocytes. (**A**) Quantification of IL-1α (n = 4) present in the KC SLGN-CM (Mann-Whitney test). (**B**) Experimental protocol for the treatment of PHH with SLGN-CM (1/50) or IL-1α at concentrations present in the SLGN-CM (<5 pg/mL after 1/50 dilution) in combination with IL-1α-neutralizing antibody for 72 h prior HBV inoculation (6 d). (**C-G**) Treatment of PHH with an IL-1α-neutralizing antibody prior HBV infection does not prevent the decrease in HBsAg, HBeAg, 3.5-kb RNA and cccDNA levels observed with SLGN-CM alone (One-way ANOVA, n = 3). Bars represent mean ± SEM. cccDNA, covalently closed circular DNA; CM, conditioned media; HBeAg, hepatitis B e antigen; HBsAg, hepatitis B surface antigen; HBV, hepatitis B virus; IL, interleukin; KCs, Kupffer cells; PHH, primary human hepatocytes; SLGN, Selgantolimod.

Antibody name:	Usage:	Reference:
Anti-p-STAT3 Y705	WB: 1/2000, TBS-T, overnight incubation	9145, Cell Signaling Technology
Anti-total STAT3	WB: 1/1000, TBS-T, overnight incubation	12640, Cell Signaling Technology
Anti-β-actin	WB: 1/1000, TBS-T, overnight incubation	3700, Cell Signaling Technology
Anti-TLR8	WB: 1/1000, TBS-T, overnight incubation	11886, Cell Signaling Technology
Anti-rabbit HRP	WB: 1/5000, TBS-T, 1 h incubation	12-348, Sigma-Aldrich
Anti-mouse HRP	WB: 1/5000, TBS-T, 1 h incubation	12-349, Sigma-Aldrich
Anti-CD163	IF: 5 µg/mL, overnight incubation	ab87099, Abcam
Anti-CD68	IF: 1 µg/mL, overnight incubation	ab955, Abcam
Anti-rabbit Alexa488	IF: 1/1000, 1 h incubation	4412, Cell Signaling Technology
Anti-mouse Alexa594	IF: 1/800, 1 h incubation	A-11032, Invitrogen
Rabbit IgG Ctrl	IF: 5 µg/mL, overnight incubation	ab171870, Abcam
Mouse IgG Ctrl	IF: 1 µg/mL, overnight incubation	15-6E10A7, Abcam
Anti-RNA Pol2	ChIP: 2 µg/reaction, overnight incubation	91151, Active Motif
Anti-SP2 RNA Pol2	ChIP: 2 µg/reaction, overnight incubation	91115, Active Motif
Anti-SP5 RNA Pol2	ChIP: 2 µg/reaction, overnight incubation	91119, Active Motif
Mouse IgG Ctrl	ChIP: 2 µg/reaction, overnight incubation	C15400001-15, Diagenode
Anti-IL-6	NEU: 1 µg/mL	MAB206, R&D Systems
Anti-OSM	NEU: 1 µg/mL	MAB295, R&D Systems
Anti-IL-24	NEU: 1 µg/mL	AF1965, R&D Systems
Anti-IL-1α	NEU: 1 µg/mL	MAB200, R&D Systems
Mouse IgG Ctrl	NEU: 1 µg/mL	MAB002, R&D Systems

Supplemental table S1. List of antibodies. ChIP, chromatin immunoprecipitation; HRP, horseradish peroxidase; IF, immunofluorescence; Ig, immunoglobulin; IL, interleukin; NEU, neutralization; OSM, oncostatin M; Pol2, RNA polymerase 2; STAT3, signal transducer and activator of transcription 3; WB, western blot; TLR8, Toll-like receptor 8.

Primer name:	Sequence (5' -> 3'):
GAPDH-FW	TGCACCACCAACTGCTTA
GAPDH-BW	GGATGCAGGGATGATGTTC
EREG-FW	CACAGTCGTCGGTTCCACA
EREG-BW	ACCAGGCACACTGTTATCCC
S100A12-FW	TTCCTGTGCATTGAGGGGTT
S100A12-BW	TGTCAAAATGCCCCTTCCGA
TIMD4-FW	ATTGAGTTTGGTGGCTTTACCT
TIMD4-BW	GAGTAGGGGCACTGGTCTTTC
FOLR2-FW	CCTGTACCGAAGACAGAGGC
FOLR2-BW	GAGCTGAACCTCCGTTGCT
CD5L-FW	GTGGGTCGAATGTGAAGATCC
CD5L-BW	CATAGCATTTCCGGTCTCTGAAG
NR1H3-FW	CCTTCAGAACCCACAGAGATCC
NR1H3-BW	ACGCTGCATAGCTCGTTCC
SPIC-FW	ATGACGTGTGTTGAACAAGACA
SPIC-BW	CGATGGTTGATTAAAGCCAGGT
PDL1-FW	GGCATTTGCTGAACGCATTTACT
PDL1-BW	AGTGCAGCCAGGTCTAATTGT
NTCP-FW	CATAGGGATCGTCCTCAAATCCA
NTCP-BW	GCCACACTGCACAAGAGAATG
SOCS3-FW	CGACGGGACCTTCTTGTTG
SOCS3-BW	GTCACTGCGCTCCAGTAGAA
NTCP-Pro-FW	TGACAAGGGAGGAGTACAAGTAGCACCCAG
NTCP-Pro-BW	CCTCCTGTGAGGCAGTGGAAGACCACTCC
cccDNA-FW	CCGTGTGCACTTCGCTTCA
cccDNA-BW	GCACAGCTTGGAGGCTTGA
cccDNA probe	(6FAM)CATGGAGACCACCGTGAACGCCC(BBQ)
HBB	Hs00758889_s1 (Thermo Fisher Scientific)
3.5-kb-FW	GGAGTGTGGATTCGCACTCCT
3.5-kb-BW	AGATTGAGATCTTCTGCGAC
3.5-kb probe	(6FAM)AGGCAGGTCCCCTAGAAGAAGAACTCC(BBQ)
GUSB	Hs99999908_m1 (Thermo Fisher Scientific)

Supplemental table S2. Primer sequences. cccDNA, covalently closed circular DNA; CD5L, CD5 molecule like; EREG, epiregulin; FOLR2, folate receptor beta; GAPDH, glyceraldehyde 3-phosphate dehydrogenase; GUSB, glucuronidase beta; HBB, hemoglobin subunit beta; NR1H3, nuclear receptor subfamily 1 group H member 3; NTCP, sodium/taurocholate cotransporting polypeptide; PDL1, programmed cell death 1 ligand 1; S100A12, S100 calcium binding protein A12; SOCS3, suppressor of cytokine signaling 3; SPIC, Spi-C transcription factor; TIMD4, T cell immunoglobulin and mucin domain containing 4.

SUPPLEMENTAL REFERENCES:

1 Allweiss L, Testoni B, Yu M, *et al.* Quantification of the hepatitis B virus cccDNA: evidence-based guidelines for monitoring the key obstacle of HBV cure. *Gut*. 2023;gutjnl-2022-328380.

2 Combes A, Camosseto V, N'Guessan P, *et al.* BAD-LAMP controls TLR9 trafficking and signalling in human plasmacytoid dendritic cells. *Nat Commun*. 2017;8:913.

3 Menne S, Tumas DB, Liu KH, *et al.* Sustained efficacy and seroconversion with the Toll-like receptor 7 agonist GS-9620 in the Woodchuck model of chronic hepatitis B. *J Hepatol*. 2015;62:1237–45. doi: 10.1016/j.jhep.2014.12.026

4 Trapnell C, Roberts A, Goff L, *et al.* Differential gene and transcript expression analysis of RNA-seq experiments with TopHat and Cufflinks. *Nat Protoc*. 2012;7:562–78.

5 Ramachandran P, Dobie R, Wilson-Kanamori JR, *et al.* Resolving the fibrotic niche of human liver cirrhosis at single-cell level. *Nature*. 2019;575:512–8. Gene Expression Omnibus (GEO), September 12, 2019. Data accession: GSE136103. https://doi.org/10.1038/s41586-019-1631-3

6 Hao Y, Hao S, Andersen-Nissen E, *et al.* Integrated analysis of multimodal single-cell data. *Cell*. 2021;184:3573-3587.e29.

7 Korsunsky I, Millard N, Fan J, *et al.* Fast, sensitive and accurate integration of single-cell data with Harmony. *Nat Methods*. 2019;16:1289–96. doi: 10.1038/s41592-019-0619-0

8 Han L, Wei X, Liu C, *et al.* Cell transcriptomic atlas of the non-human primate Macaca fascicularis. *Nature*. 2022;604:723–31. Non-Human Primate Cell Atlas. https:// doi.org/10.1038/s41586-022-04587-3

9 Hänzelmann S, Castelo R, Guinney J. GSVA: gene set variation analysis for microarray and RNA-Seq data. *BMC Bioinformatics*. 2013;14:7.
